# The inactivated herpes zoster vaccine HZ/su induces a varicella zoster virus specific cellular and humoral immune response in patients on dialysis

**DOI:** 10.1016/j.ebiom.2024.105335

**Published:** 2024-09-11

**Authors:** Franziska Hielscher, Tina Schmidt, Martin Enders, Sarah Leyking, Markus Gerhart, Kai van Bentum, Janine Mihm, David Schub, Urban Sester, Martina Sester

**Affiliations:** a Department of Transplant and Infection Immunology, Saarland University, Homburg, Germany; b Labor Enders und Partner, Stuttgart, Germany; c Praxis Dr. Leyking, St. Ingbert, Germany; d Nieren- und Dialysezentrum St. Wendel, St. Wendel, Germany; e Heimdialyse Saar e.V. Dialysezentrum Homburg, Homburg, Germany; f Department of Nephrology, SHG-Klinikum Völklingen, Germany; g Center for Gender-specific Biology and Medicine (CGBM), Saarland University; Homburg, Germany

**Keywords:** Patients on dialysis, Herpes zoster, Vaccination, Varicella zoster virus, T-cells, Antibodies

## Abstract

**Background:**

To evaluate the immunogenicity of the inactivated herpes-zoster vaccine HZ/su in patients at increased risk for VZV-reactivation, we analysed the quantity and quality of the vaccine-induced cellular and humoral immunity in patients on dialysis with uremic immunodeficiency.

**Methods:**

In this observational study, 29 patients and 39 immunocompetent controls underwent standard dual-dose vaccination. Blood samples were analysed before and two weeks after each vaccination, and after one year. Specific T-cells were characterized after stimulation with VZV-gE-peptides based on induction of cytokines and CTLA-4-expression using flow-cytometry. Antibodies were analysed using ELISA.

**Findings:**

Both groups showed an increase in VZV-gE-specific CD4 T-cell levels over time (p < 0.0001), although median levels reached after second vaccination were lower in patients (0.17% (IQR 0.21%)) than in controls (0.24% (IQR 0.3%), p = 0.042). VZV-gE specific CD8 T-cells were only poorly induced. CTLA-4 expression on VZV-gE-specific CD4 T-cells was strongest after second dose with no differences between the groups (p = 0.45). Multifunctional cells co-expressing IFNγ, IL-2, and TNF were higher in patients after first vaccination (p = 0.028). Median VZV-specific IgG-levels reached a maximum after second vaccination with significantly lower levels in patients (10796 (IQR 12482) IU/l) than in controls (16899 (IQR 14019) IU/l, p = 0.009). Despite similar CD4 T-cell levels after one year (p = 0.415), antibody levels remained significantly lower in patients (p = 0.0008).

**Interpretation:**

VZV-gE vaccination induced specific antibodies and CD4 T-cells in both patients and controls, whereas CD8 T-cell-induction was poor. Quantitative and qualitative differences in immunity may indicate reduced duration of protection which may necessitate booster vaccinations in patients on dialysis.

**Funding:**

HOMFORexzellent (to D.S.).

**Research in context**

**Evidence before this study**

To date, knowledge about the safety and immunogenicity of the inactivated HZ/su vaccine in patients on dialysis with uremic immunodeficiency compared to immunocompetent controls is limited. We searched Pubmed without language restriction for studies on the safety and immunogenicity of the recombinant HZ/su vaccine in patients on dialysis. Terms for systematic search were “recombinant zoster vaccine dialysis or CKD” or “recombinant zoster vaccine immunogenicity” or “HZ/su vaccine immunogenicity” or “recombinant zoster vaccine immunocompromised” or “HZ/su T cells” or “HZ/su antibodies”. No articles or reports on humoral or cellular immunogenicity of HZ/su were found for patients on dialysis. After initiating our study, we found one trial, where immunogenicity was analysed in the context of a phase 3, randomized (1:1) study among 264 renal transplant recipients who received 2 doses of the recombinant zoster vaccine HZ/su or placebo 1–2 months apart. Vaccine-induced antibodies were analysed prior to vaccination, 1–2 months after the first dose, as well as 1, 6 and 12 months after the second dose. The adjusted anti-gE antibody geometric mean concentration (GMC) ratio (HZ/su over placebo) was 14.00 (95% CI, 10.90–17.99; p < 0.0001) 1 month after the second dose. Cell-mediated immunity (CMI) was tested in a subset of 64 patients prior to vaccination as well as 1 and 12 months after the second dose. The geometric mean ratio (HZ/su over placebo) of gE-specific CD4 T-cell frequencies was 17.26 (95% CI, 5.92–50.36; p < 0.0001) after the second dose. Median CD4 T cell levels in the HZ/su group decreased by month 12 but remained higher than prior to vaccination. In contrast, antibody- and T-cell levels remained low in the placebo group. In the meantime, two more studies on solid organ transplant patients were published. Both studies showed a significant increase in anti-gE antibody levels and an induction of gE-specific polyfunctional CD4 T-cell frequencies after the first and second vaccine doses. However, no follow-up analyses or immunocompetent individuals were carried out in parallel, which does not allow to address the contribution of immunodeficiency on the induction and maintenance of the immune response.

**Added value of this study**

In this study, we characterized the safety as well as cellular and humoral immune response induced by HZ/su in patients on dialysis compared to immunocompetent individuals. The vaccine was well tolerated and induced VZV-specific CD4 T-cells and antibodies in both controls and patients on dialysis, whereas VZV-specific CD8 T-cells were only poorly induced. VZV-specific CD4 T-cells were multifunctional and showed a dynamic increase with a maximum after the second vaccination. However, median T-cell levels were lower in patients as compared to controls. Likewise, VZV-specific IgG antibodies showed a dynamic increase in both groups, although antibody levels remained lower in patients compared to controls.

**Implications of all the available evidence**

Two doses of the HZ/su induced VZV-specific humoral and cellular immune response in patients on dialysis and were well tolerated. Nevertheless, the quantity and quality of vaccine-induced VZV-specific T-cells and lower antibody levels in patients may suggest a reduced protective effect, which may indicate the need for more frequent booster vaccinations in this vulnerable patient group.

## Introduction

Varicella zoster virus (VZV), a member of the alphaherpesvirus family, causes chickenpox during primary infection in childhood, and then remains latent in dorsal root ganglia or cranial nerves. VZV may reactivate from latency causing herpes zoster (HZ),^1^ which typically manifests as a painful, dermatomal vesicular rash and may lead to serious complications such as postherpetic neuralgia (PHN).^2^^,^^3^ The probability of reactivation depends on the individual's immunity, whereby reduced VZV-specific T-cell levels are considered to be the main determinant. A decline in VZV-specific T-cell immunity with increasing age is associated with an increased risk of VZV reactivation among the elderly.^4^^,^^5^ The incidence of HZ is about 4–4.5 per 1000 person-years and increases to more than 11 per 1000 person-years in people aged 80 years or older.^6^ Another factor that favors VZV reactivation and increases the risk of herpes zoster is an impaired immune system.^7^^,^^8^ Patients on dialysis with uremic immune dysfunction also belong to this risk group, which has a higher probability of VZV reactivation than immunocompetent individuals.^9^^,^^10^ These patients often exhibit a disturbed interaction between APCs and T-cells and an increased production of pro-inflammatory cytokines.^11^ This is associated with more frequent and severe infections^12^ as well as an inadequate vaccination response, e.g. against hepatitis B, tetanus,^13^ diphtheria^14^ and influenza.^15^^,^^16^

In 2018, a recombinant glycoprotein E (gE) subunit herpes zoster vaccine (HZ/su) was approved in Germany.^17^^,^^18^ Unlike the previously available live-attenuated vaccine Zostavax, HZ/su is also suitable for immunocompromised people for whom the live-attenuated vaccine is normally contraindicated.^19^^,^^20^ HZ/su consists of VZV-gE and AS01_B_ as adjuvant system, which contains *Quillaja saponaria* Molina, fraction 21 (QS-21) and 3-O-desacyl-4′-monophosphoryl lipid A (MPL) from *Salmonella Minnesota*.^21^ It has already been shown to be effective for healthy individuals^22^^,^^23^ or patients after autologous stem cell transplantation.^24^ Nevertheless, we hypothesized that immunogenicity may be impaired in immunocompromised patients. First immunogenicity data exist in patients after solid organ transplantation.^25^, ^26^, ^27^ However, a direct comparison with controls was not performed, and knowledge on the vaccine-induced immune response and its stability in patients on dialysis is limited. In this observational study, we therefore aimed at characterizing the reactogenicity and immunogenicity of HZ/su in patients on dialysis compared to healthy individuals. In addition to the quantitative determination of VZV-specific T-cells and antibodies, a qualitative investigation of proliferative capacity, cytokine expression of vaccine-induced T-cells and neutralizing effect of the specific antibodies was performed.

## Methods

### Recruitment of the study population

Patients on dialysis and immunocompetent age-matched controls without restriction for sex/gender and without history of herpes zoster vaccination receiving two standard dosages of the HZ/su vaccine based on standard recommendations were enrolled in an observational study from 06/2019 to 12/2021. Individuals were recruited from multiple centers in the German federal state of Saarland. Patients had been on dialysis for at least 6 months. Whole blood samples were collected before the first and second HZ/su vaccination, two weeks after each vaccine dose as well as 12 months after the first (i.e. 9 months after the second vaccination; Supplementary Figure S1). Blood samples were drawn before the dialysis sessions. Study participants completed a questionnaire in which they self-reported their recall of previous chickenpox infections or herpes zoster, as well as local and systemic adverse events occurring within the first week after each vaccination. We anticipated a sample size of approximately 30 individuals per group based on previous immunogenicity studies.^15^^,^^28^ The study was approved by the ethics committee of the Ärztekammer des Saarlandes (reference 27/19), and all individuals gave written informed consent. The funder (HOMFORexzellent) had no role in the design or analysis of the study.

### Quantification of lymphocyte subpopulations

To quantitate lymphocyte subpopulations, 100 μl heparinized whole blood was washed once with RPMI, stained for characteristic phenotypic markers and analysed using flow cytometry as described before.^29^ To identify follicular helper T-cells, antibodies against CD4 (clone SK3, 1:5.5) and CXCR5 (clone RF8B2, 1:5.5) were used. In addition, T-cells were analysed for the surface markers inducible costimulator (ICOS; clone DX29, 1:3.65) and programmed cell death protein 1 (PD-1; clone MIH4, 1:3.65). B-cells were identified as CD19^+^ (clone HIB19, 1:16.7) CD3^-^ (clone SK7, 1:26.7) and their differentiation status was determined using antibodies against IgD (clone IA6-2, 1:20) and CD27 (clone L128, 1:3.3). Among switched-memory B-cells, plasmablasts were identified as CD38^+^ (clone HB-7, 1:33.3). Antibodies including Research Resource ID (RRID) are listed in Supplementary Table S2.

### Quantification and characterization of VZV-specific T-cells

The analysis of antigen-specific T-cells was carried out as previously described for other VZV antigens.^30^^,^^31^ In brief, 450 μl heparinized whole blood was stimulated with 2 μg/ml overlapping VZV gE peptides (Swiss-Prot ID: P09259, JPT, Berlin, Germany). As a positive control, blood samples were stimulated with 2.5 μg/ml *Staphylococcus aureus* enterotoxin B (SEB) (Sigma–Aldrich, St. Louis, MO, USA), while 0.56% DMSO served as a negative control. All stimulations were performed in the presence of 1 μg/ml anti-CD28 and anti-CD49d antibodies (BD Biosciences, San Jose, CA, USA). After 2 h of incubation, 10 μg/ml brefeldin A was added for intracellular cytokine accumulation. After an additional 4 h, cells were fixed and immunostaining was performed after permeabilization using anti-CD4 (clone SK3, 1:33.3), anti-CD8 (clone SK1, 1:12.5), anti-CD69 (clone L78, 1:33.3), anti-interferon (IFN) γ (clone 4S.B3, 1:100), anti-interleukin 2 (IL-2) (clone MQ1-17H12, 1:16.7), anti-tumor necrosis factor (TNF; clone MAb11, 1:20), and anti-cytotoxic T-lymphocyte-associated protein 4 (CTLA-4; clone BNI3, 1:50). To characterize VZV-specific memory T-cells, immunostaining was performed using anti-CD4 (clone SK3, 1:100), anti-CD69 (clone L78, 1:25), anti-IFNγ (clone 4S.B3, 1:100), anti-CD45RO (clone UCHL-1, 1:40), and anti-CD27 (clone M-T271, 1:20). Antibodies including RRID are listed in Supplementary Table S3. Flow cytometric analyses were performed on FACS Canto II, using FACSDiva Software 6.1.3 (BD). VZV-specific CD4 or CD8 T-cells were identified as activated CD69-positive T-cells producing IFNγ and further characterized for expression of cell surface markers and additional cytokines. Percentage of VZV-specific CD4 and CD8 T-cells were determined by subtracting the corresponding negative controls.

### Proliferation activity of VZV-specific T-cells

VZV-specific proliferation was analysed using carboxyfluoresceindiacetate-succinimidylester (CFDA-SE) assay, as described before.^32^ In brief, peripheral blood mononuclear cells (PBMC) were isolated by Ficoll density gradient (Linaris) and stained with CFDA-SE (5 μM, Invitrogen). Cells were cultured at 2 × 10^7^ cells/ml in RPMI-5% FCS-1% antibiotics in the presence of 2 μg/ml overlapping VZV gE peptides (Swiss-Prot ID: P09259, JPT, Berlin, Germany). Negative or positive control stimulations were carried out using 0.56% DMSO or 2.5 μg/ml SEB, respectively. Cells were incubated at 37 °C and 5% CO2. SEB stimulated cells were splitted (1:1) on day 3 or 4. Flow cytometric analysis was performed after 7 days of proliferation after co-staining using antibodies towards CD3 (clone SK7, 1:50), CD4 (clone SK3, 1:12.5), CD8 (clone SK1, 1: 8.33). Antibodies including RRID are listed in Supplementary Table S3.

### Analysis of VZV-specific IgG antibodies and neutralization activity

VZV-specific antibodies were quantified using a commercial anti-IgG enzyme-linked immunosorbent assay (ELISA; Euroimmun AG, Lübeck, Germany). IgG levels <80 international unit (IU)/L were scored negative, levels 80–110 IU/l were scored intermediate, and levels >110 IU/l were scored positive according to the manufacturer's instructions.

To analyse the functionality of VZV-specific antibodies, a neutralization test was carried out. The assay was performed in duplicate using serial dilutions of serum samples in E-MEM-2% FCS-0.1% antibiotics. Subsequently, 100 μl VZV (Clade 3, strain Nr. 13, Original-Nr: 1219/07; kindly provided by Prof. Dr. med. Hartmut Hengel; reference laboratory for HSV/VZV; Freiburg; Germany) diluted 1:40 in medium was added and the samples were incubated for 90 min at 37 °C and 5% CO_2_. Finally, 100 μl embryonic lung fibroblasts (100,000 cells/ml) were added and plates were incubated for 5 days. Serial dilutions without serum served as positive control and a dilution series with cell suspension only served as negative control. The cells were fixed for 10 min with 100 μl ice-cooled acetone/methanol (40:60). Anti-VZV antibody towards the immediate early gene 62 (MAB 8616, 1:1000, Sigma–Aldrich) was added as primary antibody. Goat anti-Mouse IgG (1:200, Thermo Scientific) was used as secondary antibody. After adding 50 μl AEC substrate (Sigma–Aldrich) and incubating for 30 min at 37 °C and 5% CO_2_, the reaction was stopped with distilled water. Finally, plaques were counted microscopically, and the geometrical mean (GM) was determined.

### Statistical analysis

The Mann–Whitney test was used for analysis of data between two groups. Longitudinal analyses of paired samples were performed using the Friedman test with Dunn's post-test or the Wilcoxon test. Fisher's exact test and Chi-square test was used to analyse differences in categorical variables. Correlations were calculated using the nonparametric Spearman test. A two-way ANOVA (Mixed effects analysis with Tukey's post test) was used to compare vaccine-induced parameters over time in patients and controls. Statistical analysis was performed using GraphPad-Prism-V9.2.0 (GraphPad, San Diego, CA). A p-value <0.05 was considered statistically significant.

### Role of funders

The funder did not have any role in study design, data collection, data analyses, interpretation, or writing of report.

## Results

### Study population

A total of 29 patients on dialysis and 39 healthy controls were recruited, who received the dual dose inactivated HZ/su vaccine. Demographic characteristics are shown in Table 1, with underlying diseases of patients on dialysis listed in Supplementary Table S1. There was no difference in age and sex between the patients and controls. Most subjects reported a history of chickenpox (controls 91.2%; patients 76.2%), and 24.0% of patients and 18.4% of controls had already suffered from herpes zoster. Patients and controls differed in their leukocyte subpopulations (Table 1). Monocyte and granulocyte counts were significantly higher in patients on dialysis (p = 0.006 and p = 0.035, respectively), whereas lymphocyte counts were significantly lower (p < 0.0001). While CD8 T-cell counts did not differ in both groups, patients on dialysis had significantly lower CD4 T-cell counts (p = 0.031). In addition, numbers of CD19-positive B-cells (p < 0.0001) and plasmablasts (p = 0.004), identified as CD38-positive cells among IgD^−^CD27^+^ CD19-positive switched-memory B-cells, were significantly lower in patients on dialysis.

**Table 1:** Demographic and basic characteristics of the study populations.

	Patients on dialysis^b^	Controls	p-value
	29	39	
**Years of age (mean ± SD)**	70.3 ± 11.7	67.9 ± 8.2	0.334^e^
**Sex**^a^**, n (%)**			
Female	12 (41.4)	24 (61.5)	0.141^e^
Male	17 (58.6)	15 (38.5)	
**Years on dialysis, mean ± SD**	4.81 ± 4.57	n.a.	
**History of chickenpox**^c^**, n (%)**	16 (76.2)	31 (91.2)	0.236^e^
**History of herpes zoster**^c^**, n (%)**	6 (24.0)	7 (18.4)	0.752^e^
**Weeks between 1**st **and 2**nd **vaccination, mean ± SD**	15.5 ± 2.9	13.3 ± 4.1	
**Analysis time [days after 1**st **vaccination], median (IQR)**	14 (0)	14 (0)	
**Analysis time [days after 2**nd **vaccination], median (IQR)**	14 (0)	14 (0.25)	
**Analysis of follow-up**			
Yes	20	38	
No (COVID-19 infection, lockdown)	1	1	
No (transplanted)	1	n.a.	
No (died)	7	0	
**Differential blood cell counts, median (IQR) cells/μl**	n = 29	n = 38	
Leukocytes	6200 (2395)	6150 (1530)	0.394^d^
Granulocytes	4736 (2221)	3713 (1256)	0.035^d^
Monocytes	650 (328)	471 (183)	0.006^d^
Lymphocytes	1222 (750)	1811 (855)	0.0001^d^
CD3 T-cells	935 (524)	1276 (711)	0.005^d^
CD4 T-cells	659 (369)	926 (410)	0.031^d^
CD8 T-cells	260 (166)	279 (250)	0.410^d^
CD19 B-cells	81 (92)	173 (133)	<0.0001^d^
Plasmablasts	0.224 (0.562)	0.488 (0.498)	0.004^d^
Follicular T helper cells	108.7 (67.24)	105 (64.59)	0.674^d^

a Self-reported.

b 28 patients on hemodialysis, 1 patient on lipid apheresis.

c Self-reported information to remember having had a history of chickenpox or herpes zoster.

d Mann–Whitney *U* test.

e Fisher's exact test.

### HZ/su vaccine mainly induces VZV-specific CD4 T-cells with only low levels of CD8 T-cells

**Fig. 1:**
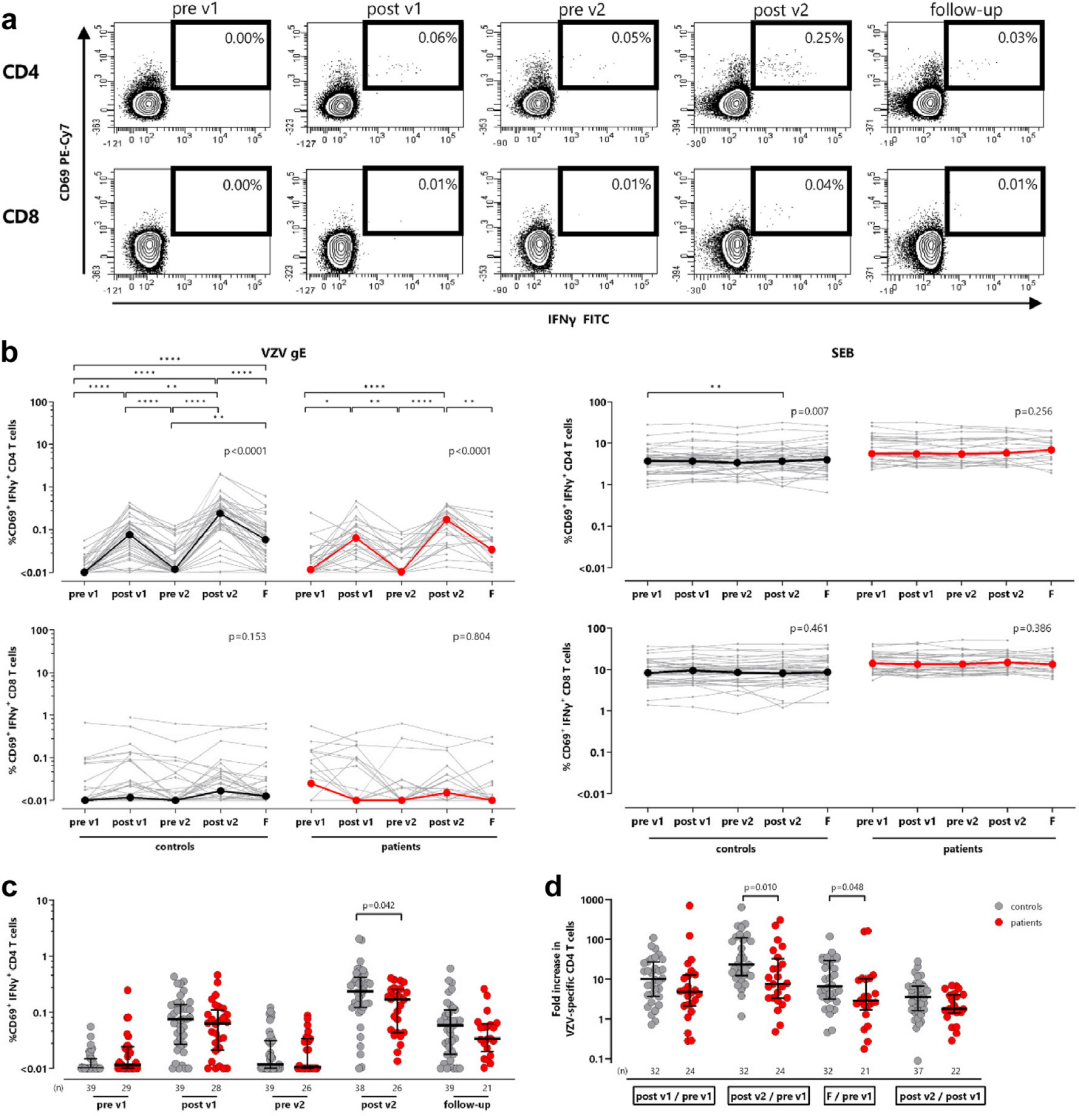
**VZV-specific CD4 T-cell levels increase after HZ/su vaccination**. **(a)** Representative contour plots of specific CD4 and CD8 T-cells of a male dialysis patient in his fifties before the first and the second vaccinations (pre v1; pre v2), two weeks after the first and the second vaccinations (post v1; post v2) as well as 9 months after the second vaccination (follow-up) determined after stimulation of whole blood with overlapping peptides of VZV gE. Numbers indicate the percentages of reactive CD4 and CD8 T-cells defined by co-expression of the activation marker CD69 and the cytokine IFNγ. **(b)** Percentages of VZV-specific CD4 (upper panels) and CD8 T-cells (lower panels) after subtraction of the corresponding negative control (left) and SEB-reactive CD4 and CD8 T-cells (right) over time. Bold lines represent median values. Friedman test with Dunn's post test was performed for statistical analysis. Tabular results of differences in the time course between patient and controls are shown in Supplementary Table S2. **(c)** Comparison of samples from healthy controls (grey) and patients on dialysis (red) at each time point. Bars represent median values with interquartile ranges. **(d)** For each individual the fold increase in VZV-gE specific CD4 T-cell levels was calculated after the first and second vaccination and at follow-up compared with baseline (pre v1) and between the first and the second vaccination (post v1/pre v1, post v2/pre v1, follow-up/pre v1 and post v2/post v1). Statistical analysis in (c) and (d) was performed using Mann–Whitney test. Absolute numbers of VZV-specific and SEB-reactive CD4 T cells are shown in Supplementary Figure S2. Sex-disaggregated data are shown in Supplementary Figures S6 and S7. F, follow-up; IFN, interferon; VZV, *Varicella zoster virus*; SEB, *Staphylococcus aureus* enterotoxin B.

The induction of the VZV-specific immune response by the HZ/su vaccine was analysed in patients and controls immediately before each vaccination (pre v1 and pre v2), 2 weeks after (post v1 and post v2) and 12 months after the first vaccination (follow-up, with a schematic chart shown in Supplementary Figure S1). VZV-specific T-cells were identified using flow cytometry based on the induction of the activation marker CD69 and the cytokine IFNγ after specific stimulation with overlapping VZV gE peptides. Diluent (DMSO) and *S. aureus* Enterotoxin B (SEB) was used as negative and positive control stimuli, respectively. Representative contour plots of blood samples of a male hemodialysis patient in his fifties after peptide stimulation is shown in Fig. 1a. A significant increase in vaccine-specific CD4 T-cells was observed two weeks after the first and the second vaccination in both patients and controls, with the maximum peak after the second vaccination (Fig. 1b). Differences in the time course between patients and controls are shown in Supplementary Table S3. At follow-up, VZV-specific CD4 T-cell levels decreased again, but remained higher than CD4 T-cell frequencies prior to vaccination (p < 0.0001). In contrast, neither patients nor controls showed a significant increase in VZV-specific CD8 T-cell levels after the two vaccinations. In some cases, VZV-specific CD8 T-cell levels were high even before vaccination, and remained stable over time. Despite similar dynamics in VZV-specific CD4 T-cells in both groups, patients reached significantly lower VZV-specific CD4 T-cell levels (0.17% (IQR 0.21%)) two weeks after the second vaccination compared to healthy controls (0.24% (IQR 0.3%) p = 0.042, Fig. 1c). Likewise, the median increase in the percentage of specific CD4 T-cells from baseline to two weeks after the second vaccination was lower in patients on dialysis (7.7-fold) than in controls (23.3-fold; p = 0.010). Finally, at one year follow-up, the increase in patients was significantly lower than in controls (2.9-fold versus 6.6-fold; p = 0.048, Fig. 1d). Overall, dynamics in T-cell levels were vaccine-specific, as the percentage of SEB-reactive CD4 and CD8 T-cells were largely similar in the two groups and remained stable over time (Fig. 1b). Absolute numbers of VZV-specific and SEB-reactive CD4 T-cells are shown in Supplementary Figure S2.

### Vaccine-induced changes in CTLA-4 and cytokine expression in VZV-specific CD4 T-cells

**Fig. 2:**
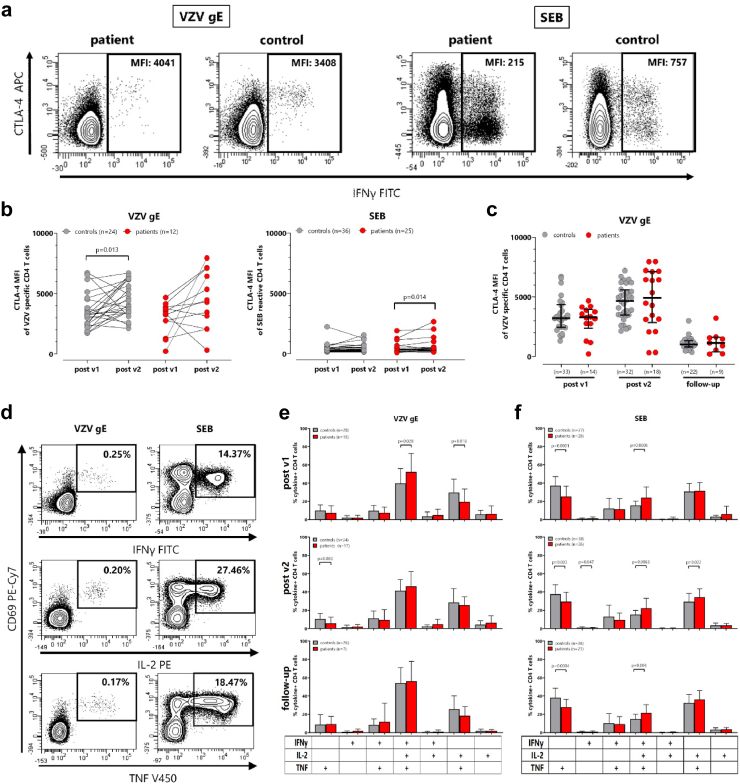
**CTLA-4 expression and cytokine profile of VZV-specific CD4 T-cells after Hz/su vaccination (a)** Representative contour plots of median fluorescence intensity (MFI) of CTLA-4 expressing VZV-specific CD4 T-cells in a male dialysis patient in his fifties and a male healthy individual in his sixties. **(b)** Comparison of CTLA-4 MFI of VZV-specific and SEB-reactive cells after the first and after the second vaccination in controls (grey, p = 0.013) and patients on dialysis (red, p = 0.064). All samples were measured. To allow for robust statistical analysis, only paired samples with at least 20 CD69^+^IFNγ^+^ CD4 T-cells were included. Differences between the time points in each group were calculated using the Wilcoxon test. **(c)** Comparison of CTLA-4 MFI on VZV-specific CD4 T-cells after both vaccinations, and on follow-up between controls and patients on dialysis. Statistical analysis was performed using the Mann–Whitney test. Tabular results of differences in the time course between patients and controls are shown in Supplementary Table S2. **(d)** Examples of contour plots of VZV-specific or SEB-reactive CD4 T-cells expressing cytokines interferon gamma (IFNγ), tumor necrosis factor (TNF) and interleukin 2 (IL-2) after stimulation of a whole blood sample from a male dialysis patient in his fifties two weeks after the second vaccination (with the contour plot showing CD69^+^IFNγ^+^ CD4 T cells corresponding to the respective plot shown in Fig. 1a). Cytokine expressing CD4 T-cells were subclassified into 7 subpopulations according to single or combined expression of IFNγ, TNF and IL-2. Blood samples from all individuals were analysed. To ensure robust statistics, only samples with at least 30 cytokine-expressing CD4 T-cells after subtraction of the corresponding negative control stimulation were considered. Comparison of cytokine profiles of **(e)** VZV-specific and **(f)** SEB-reactive CD4 T-cells in patients on dialysis and controls post v1, postv2, and on follow-up time points. Bars represent means and standard deviations. Statistical analysis was performed using the Mann–Whitney test. The final sample size is indicated in each panel. CTLA-4, cytotoxic T-lymphocyte antigen 4; IFN, Interferon; IL, Interleukin; VZV, Varicella zoster virus; SEB, *Staphylococcus aureus* Enterotoxin B.

VZV-specific CD4 T-cells were analysed for expression of CTLA-4 after both vaccinations as marker for recent antigen encounter. Contour plots of the CTLA-4 expression of VZV-specific and SEB-reactive CD4 T-cells from a male dialysis patient and a control person two weeks after the second vaccination are shown in Fig. 2a. CTLA-4 expression levels of VZV-specific CD4 T-cells were higher than of SEB-reactive CD4 T-cells (Fig. 2b). Moreover, CTLA-4 expression of VZV-specific CD4 T-cells was numerically higher after the second vaccination than after the first, although this difference only reached statistical significance in controls (p = 0.013 versus p = 0.064 in patients). CTLA-4 expression on follow-up decreased in both groups (Fig. 2c). When comparing patients and controls, no difference in CTLA-4 expression was found neither after the first nor the second vaccination (Fig. 2c).

In addition, the expression profiles of the cytokines IFNγ, IL-2 and TNF were analysed. Representative contour plots of CD69-positive VZV-specific and SEB-reactive CD4 T-cells producing the individual cytokines are shown in Fig. 2d, with quantitative analyses for all individuals displayed in Supplementary Figure S3. As with IFNγ-producing cells (Fig. 1c), the most pronounced differences between patients and controls were found after the second vaccination. Subdivision of cytokine-producing cells by boolean gating resulted in a total of seven subpopulations defined by expression of three cytokines, two cytokines or one cytokine only. VZV-specific CD4 T-cells are characterized by multifunctionality with the majority of cells simultaneously expressing all three cytokines (Fig. 2e), which contrasts with SEB-reactive CD4 T-cells, which predominantly express only TNF or TNF in combination with IL-2 (Fig. 2f). After the first vaccination, the percentage of triple positive VZV-specific CD4 T-cells was significantly higher in patients than in controls (p = 0.029), while the percentage of IL-2/TNF-expressing cells was concomitantly lower (p = 0.013). Overall, cytokine expression patterns remained similar after the second vaccination and on follow-up.

### Differentiation status of VZV-specific CD4 T-cells after vaccination

**Fig. 3:**
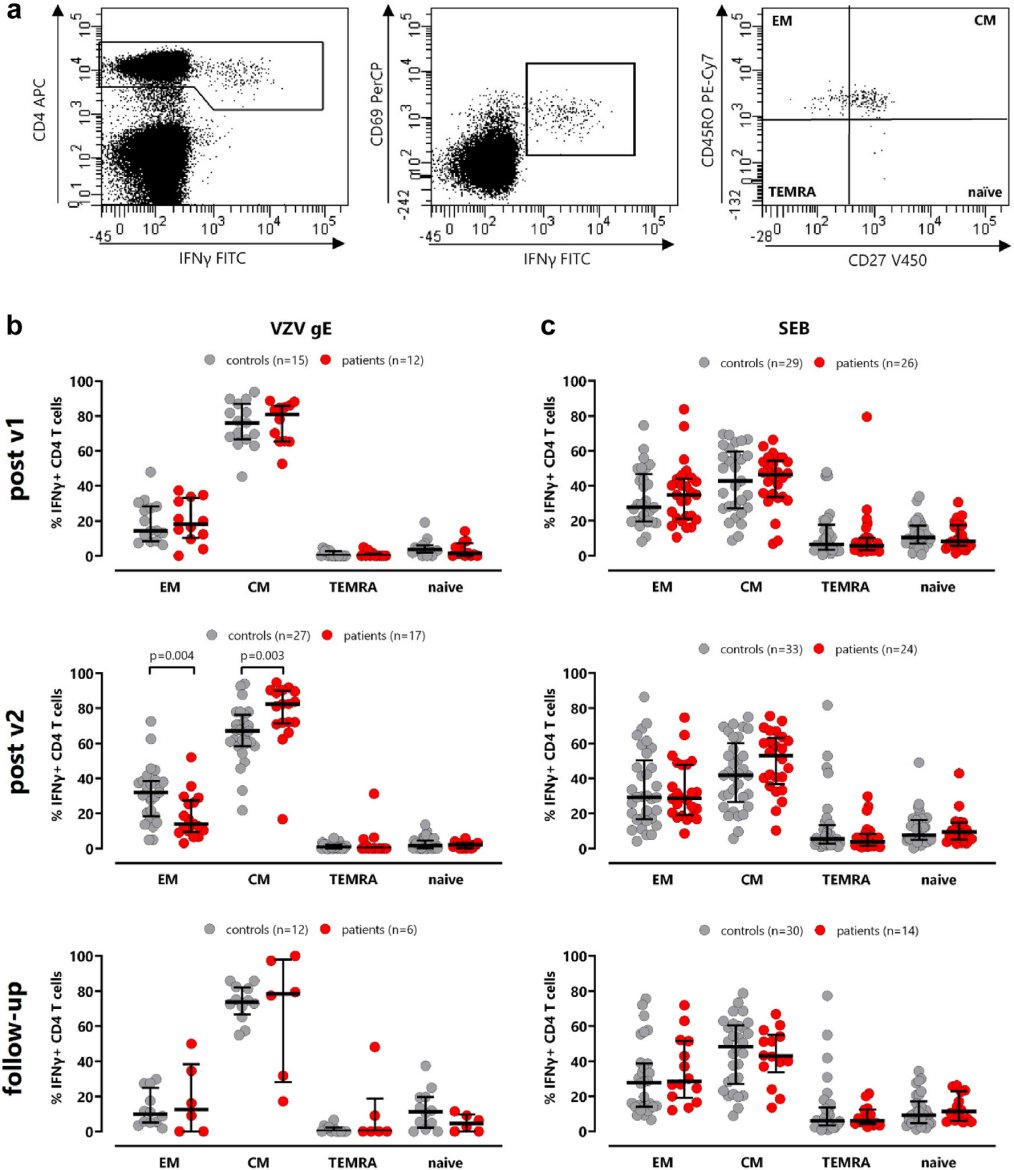
**Differences in VZV-specific CD4 differentiation between patients on dialysis and controls. (a)** Representative contour plots of the differentiation status of VZV-specific CD4 T-cells, identified by CD4 T-cells expressing CD69 and IFNγ after a 6h-stimulation with overlapping VZV gE peptides. Expression of CD45RO and CD27 was used as surrogate markers to further classify into naïve, central memory (CM), effector memory (EM), and terminally differentiated effector memory (TEMRA) cells. **(b)** T-cell populations among VZV-specific or **(c)** SEB-reactive CD4 T-cells were compared between controls (grey) and patients on dialysis (red) after the first and the second vaccinations and on follow-up. All samples were measured, but the final analysis was restricted to samples with at least 20 CD69^+^IFNγ^+^ CD4 T-cells to ensure robust statistical analysis. The final sample size is indicated in each panel. Statistical analysis was performed using Mann–Whitney test. Bars represent medians with interquartile ranges. IFN, interferon; VZV, *Varicella zoster virus*; SEB, *Staphylococcus aureus* enterotoxin B.

To analyse the differentiation status of VZV-specific CD69^+^ IFNγ^+^ CD4 T-cells, we used expression patterns of CD45RO and CD27 as surrogate for naive, central memory (CM), effector memory (EM), and terminally differentiated effector memory (TEMRA) cells after a 6h-stimulation with overlapping VZV gE peptides (Fig. 3a). After the first vaccination, CD45RO^+^CD27^+^ cells largely representing central memory cells accounted for the largest proportion of VZV-specific CD4 T-cells in both patients on dialysis (80.8% (IQR 20.3%)) and controls (76.0% (IQR 20.3%)), followed by CD45RO^+^CD27^−^ cells largely representing effector memory T-cells (patients: 18.2% (IQR 22.8%); controls 14.5% (IQR 19.8%), whereas the proportion of TEMRA or naive CD4 T-cells was very low. A similar distribution was also observed two weeks after the second vaccination and on follow-up, with some differences between patients and controls after the second vaccination. While patients had a higher percentage of central memory T-cells (p = 0.003), the percentage of effector memory T-cells was concomitantly lower than in controls (p = 0.004, Fig. 3b). Although the majority of SEB-reactive T-cells also had a central memory phenotype, the pattern was distinct from VZV-specific CD4 T-cells with no differences between patients and controls (Fig. 3c). Moreover, the distribution of VZV-specific subpopulations was distinct from bulk CD4 and CD8 T-cells (Supplementary Figure S4). Among bulk CD4 T-cells, we also characterized Tfh-cells in circulation which were predominantly of a central memory phenotype, but their levels and phenotype did not show any pronounced vaccine-related changes over time (Supplementary Figure S5).

### HZ/su induced proliferation activity in VZV-specific T-cells

**Fig. 4:**
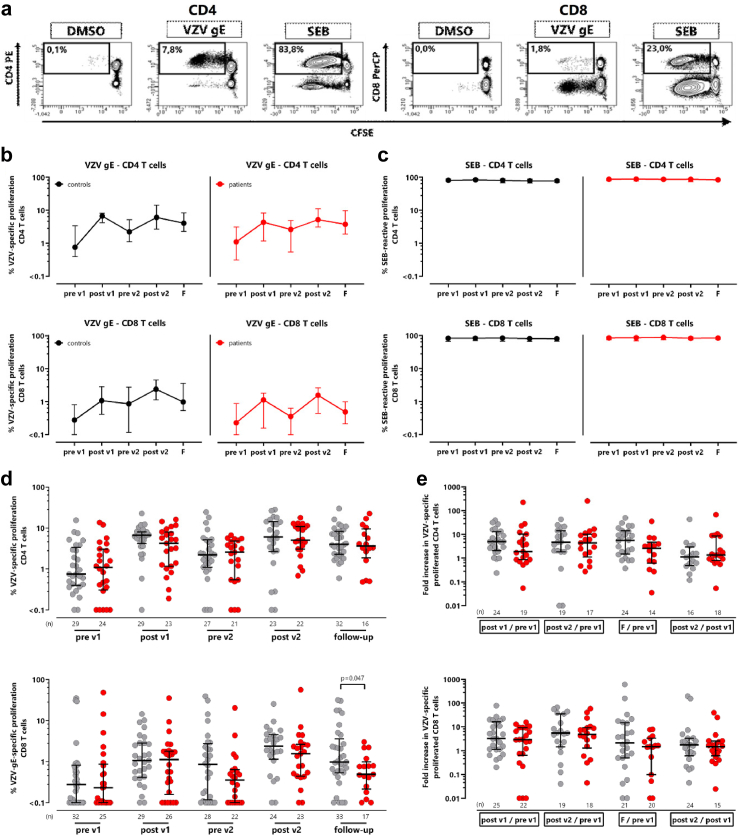
**Induction of VZV-specific T-cell proliferation in patients on dialysis after vaccination with HZ/su. (a)** Contour plots of proliferated CD4 and CD8 T-cells after staining with CFDA-SE and stimulation with overlapping VZV gE peptides, SEB (positive control) or DMSO (negative control) for 7 days. **(b)** Median percentages and IQR of proliferating VZV-specific CD4 (upper panel) or CD8 T-cells (lower panel) or **(c)** SEB-reactive CD4 or CD8 T-cells before and after vaccinations. VZV-specific CD4 and CD8 T-cells are shown after subtraction of the corresponding negative control over time in patients on dialysis (red) in comparison to healthy controls (grey). Tabular results of differences in the time course between patients and controls are shown in Supplementary Table S2. **(d)** The percentage of proliferated VZV-specific CD4 and CD8 T-cells from patients and controls are compared at each time point. **(e)** For each individual the fold increase of proliferated VZV-specific CD4 and CD8 T-cell levels was calculated after the first and second vaccination and at follow-up compared with baseline and between the first and second vaccination (post v1/pre v1, post v2/pre v1, follow-up/pre v1 and post v2/post v1). Bars refer to medians with interquartile ranges and Mann–Whitney test was used for statistical analysis. F, follow-up; VZV, *Varicella zoster virus*; SEB, *Staphylococcus aureus* enterotoxin B.

Apart from cytokine-producing cells after short-term stimulation, the proliferation capacity of VZV-specific CD4 and CD8 T-cells after antigen-specific stimulation was examined using a CFDA-SE assay. Representative contour plots of proliferating CD4 and CD8 T-cells after staining with CFDA-SE and stimulation with overlapping VZV gE peptides, DMSO diluent as negative control and SEB as positive control for 7 days are shown in Fig. 4a. Two weeks after each vaccination, an increase in the percentage of proliferating VZV-specific CD4 T-cells was observed in both patients on dialysis and controls. In addition, the proliferative capacity of VZV-specific CD4 T-cells remained higher in the follow-up samples than in samples before the first vaccination (Fig. 4b). Interestingly, VZV-specific CD8 T-cell proliferation was also induced, although the proliferative capacity was lower than that of CD4 T-cells (Fig. 4b). As expected, proliferation of SEB-reactive CD4 and CD8 T-cells was stable over time (Fig. 4c). The percentage of proliferating T-cells as well as the increase in VZV-specific T-cells were largely similar in both groups, except that the proliferative capacity of VZV-specific CD8 T-cells on follow-up was slightly lower in patients (p = 0.047, Fig. 4d and e).

### Differences in B-cell subpopulations of patients on dialysis compared to healthy individuals

**Fig. 5:**
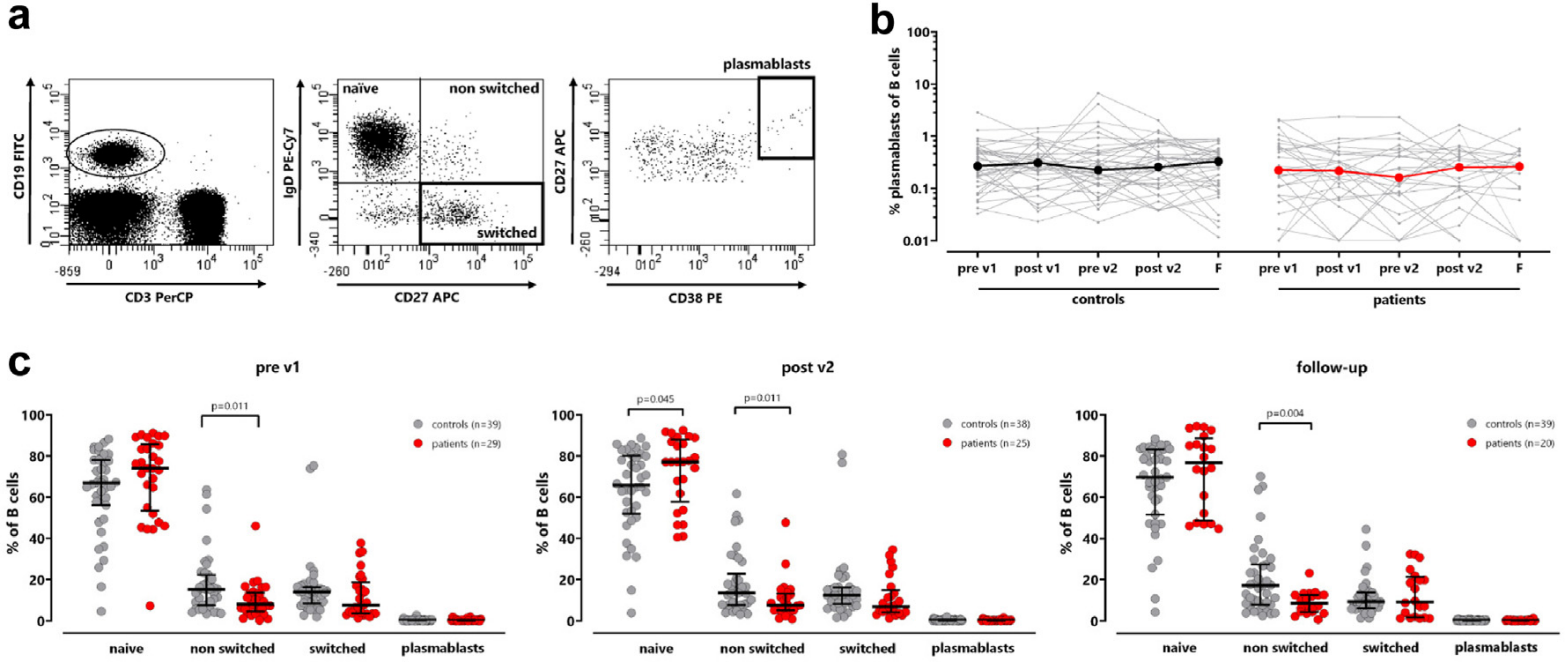
**Higher proportion of naïve B-cells in patients on dialysis. (a)** Representative dotplots of CD19+ B-cells, classified into naïve, non-switched, and switched memory B-cells based on the surface markers IgD and CD27. Plasmablasts were identified as CD38-positive cells among switched memory B-cells. **(b)** The percentage of plasmablasts was determined over time in controls (left) and patients (right). Bold lines represent median values. Tabular results of differences in the time course between patients and controls are shown in Supplementary Table S2. **(c)** B-cell subpopulations of controls (grey) and patients on dialysis (red) were compared before the first vaccination (pre v1), after the second vaccination (post v2) and on follow-up. Bars represent median values with interquartile ranges. Statistical analysis was performed using Mann–Whitney test. F, follow-up.

We next analysed B-cells and their subpopulations which were divided into naive, non-switched and switched memory B-cells based on expression of IgD and CD27. Moreover, plasmablasts were quantified as CD38-positive cells among switched memory B-cells (Fig. 5a). In both patients on dialysis and controls, the percentage of plasmablasts among B-cells was low and remained stable over time with no vaccine-associated dynamics (controls: p = 0.901; patients: p = 0.063, Fig. 5b). When analysing the distribution of the subpopulations over time, most B-cells showed a naive phenotype at all time points (Fig. 5c). Interestingly, the proportion of naive B-cells was higher in patients on dialysis than in controls, which reached statistical significance two weeks after the second vaccination (p = 0.045). In contrast, patients on dialysis showed a significantly lower percentage of non-switched B-cells compared to controls throughout the observation period (pre v1: p = 0.011; post v2: p = 0.011; follow-up: p = 0.004, Fig. 5c).

### VZV-specific humoral immune response is lower in patients on dialysis

**Fig. 6:**
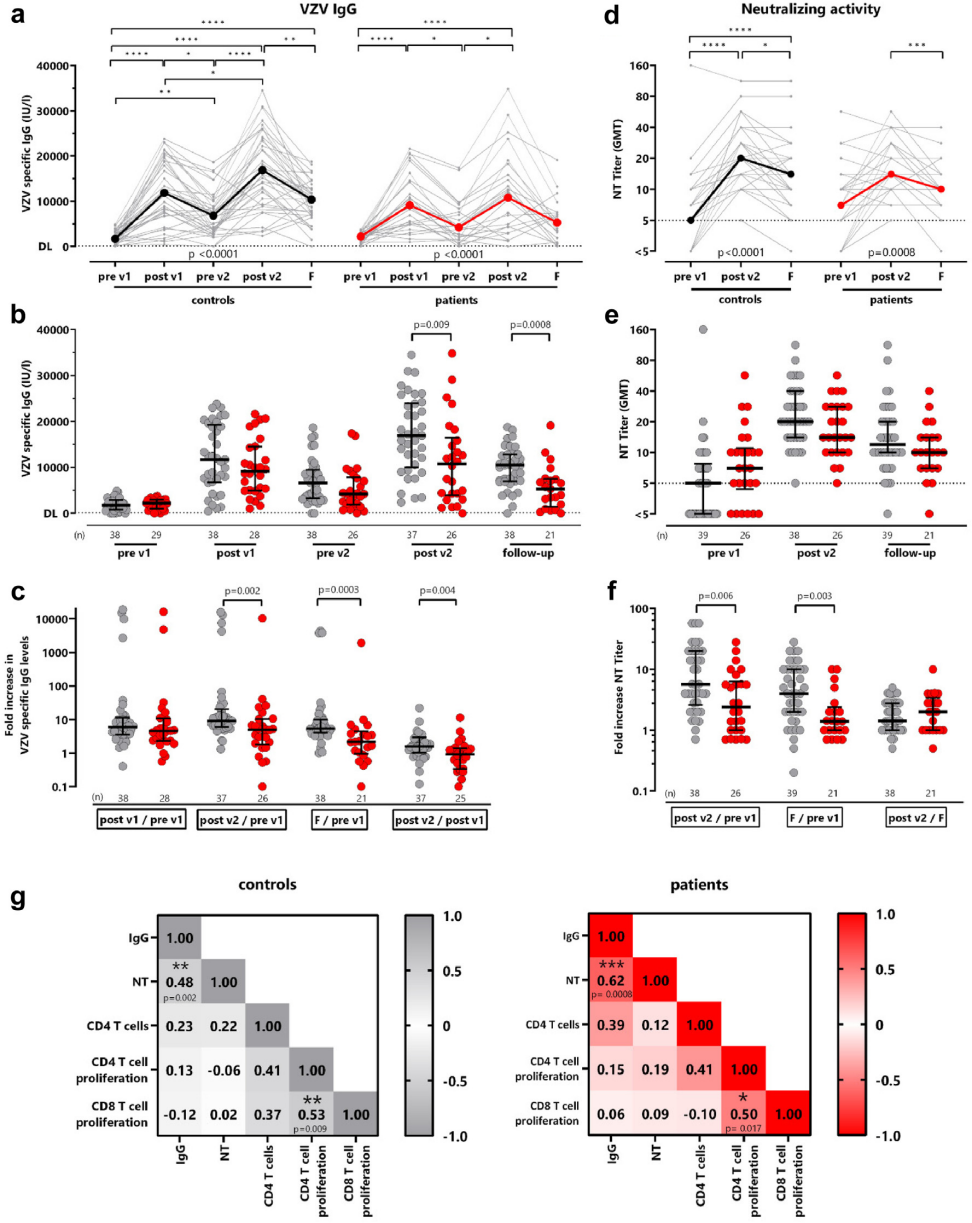
**Lower increase in VZV-specific IgG levels and neutralizing antibody titers in patients on dialysis**. **(a)** VZV IgG levels measured over time in controls (grey) and patients on dialysis (red)**. (b)** Comparison of VZV-specific IgG levels between both groups at each time point. **(c)** For each individual the fold increase in VZV-specific IgG levels was calculated after the first and second vaccination and at follow-up compared with baseline and between the first and second vaccination (post v1/pre v1, post v2/pre v1, follow-up/pre v1 and post v2/post v1). Samples <1 IU/l were set as 1 to calculate the fold increase. **(d)** VZV-specific neutralization in patients on dialysis (red) and controls (black) is shown after both vaccinations (post v1; post v2) and after one year (follow-up; F). Tabular results of differences in IgG and neutralizing titers in the time course between patients and controls are shown in Supplementary Table S2. **(e)** Comparison of neutralizing activity and **(f)** fold increase, calculated after second vaccination and follow-up compared with first vaccination and between second vaccination and follow-up, between both groups. Samples with NT titer <5 were set as 1 to calculate the fold increase. Statistical analysis of longitudinal samples was performed using the Friedman test and bold lines represent medians. Statistical analyses on the differences between the patients and controls were performed using the Mann–Whitney test, and bars represent medians with interquartile ranges. Sex-disaggregated data are shown in Supplementary Figures S6 and S7. **(g)** Correlation matrix between the percentage of vaccine-induced VZV-specific CD4 T-cells (derived from Fig. 1), IgG levels, neutralizing activity, and the percentage of proliferating VZV-specific CD4 and CD8 T-cells (derived from Fig. 4) in controls (left) and patients on dialysis (right) after second vaccination. Correlation coefficients were calculated according to two-tailed Spearman and displayed using a color code.

To investigate the vaccine-induced humoral immune response, VZV-specific IgG levels were determined by ELISA. In total, 62/68 (91.2%) of study participants were seropositive prior to vaccination, whereas only 2 patients on dialysis and 4 healthy controls had VZV-specific IgG levels below detection limit (<80 IU/l). Despite seronegativity, all 6 individuals reported a history of chickenpox. As with specific CD4 T-cells, VZV-specific IgG antibodies showed a dynamic increase in both groups (Fig. 6a). Two weeks after each vaccination, an increase in IgG concentration was observed in patients on dialysis (post v1: 9139 (IQR 9582) IU/l; post v2: 10796 (IQR 12482) IU/l) and controls (post v1: 11843 (IQR 12231) IU/l; post v2: 16899 (IQR 14019) IU/l), with peak levels reached after the second vaccination. VZV IgG levels of patients on dialysis were significantly lower than those of controls at this time point (p = 0.009). Patients on dialysis also had significantly lower VZV-specific IgG levels on follow-up (p = 0.0008, Fig. 6b). Consequently, the median increase of VZV-specific IgG from baseline to two weeks after second vaccination was also lower in patients on dialysis (5-fold) than in controls (9.1-fold; p = 0.002), as was the increase from baseline to follow-up (patients: 2.2-fold; controls: 5.4-fold; p = 0.004), or the increase from the first to the second vaccination (p = 0.0003, Fig. 6c).

Vaccination-induced neutralizing antibody titers were analysed before the first vaccination, two weeks after the second vaccination and on follow-up and reached their maximum after the second vaccination in both groups. Despite a subsequent decrease, neutralizing activity on follow-up was still higher than before the first vaccination (Fig. 6d). Although no differences in neutralizing activity were found between the two groups (Fig. 6e), patients on dialysis showed significantly less pronounced increases in neutralizing activity from baseline to two weeks after the second vaccination (p = 0.006) and from baseline to follow-up (p = 0.003, Fig. 6f).

A correlation matrix was used to investigate the relationship between the magnitudes of vaccine induced IgG, neutralizing activity, CD4 T-cells and proliferative capacity in both groups after the second vaccination. A significant correlation was observed between VZV-specific IgG levels and neutralizing activity in both controls (r = 0.48, p = 0.002) and patients (r = 0.62, p = 0.0008). Moreover, both groups showed a correlation between the proliferative capacity of VZV-specific CD4 and CD8 T-cells (controls: p = 0.009; patients: p = 0.017, Fig. 6g).

### Low reactogenicity after HZ/su vaccination in patients on dialysis

**Fig. 7:**
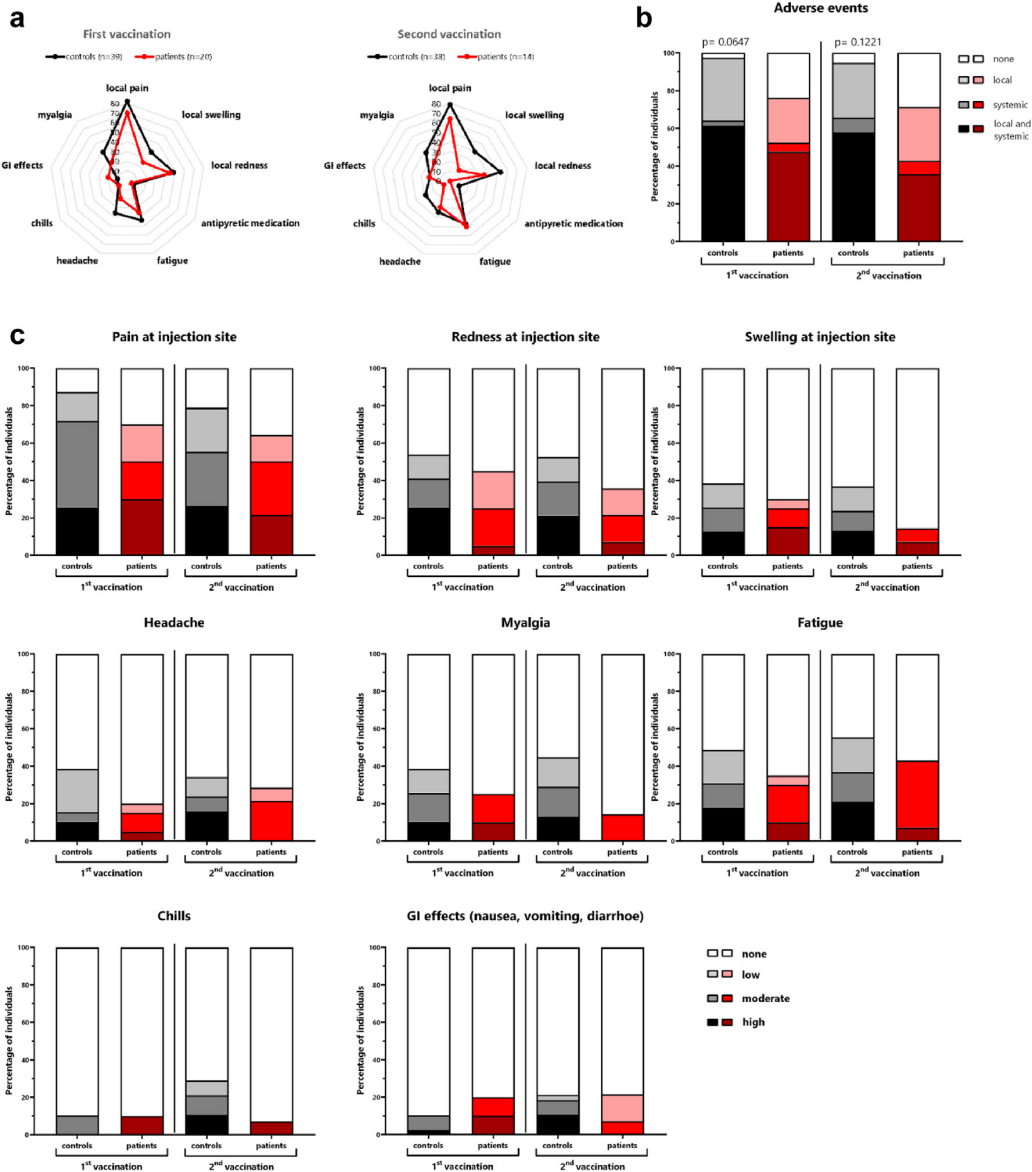
**Low reactogenicity in patients on dialysis compared to controls**. Reactogenicity within the first week after each vaccine dose was self-reported using a standardized questionnaire. **(a)** The distribution of local and systemic adverse events in healthy controls and patients on dialysis after the first and second vaccination. **(b)** Percentage of subjects who experienced no adverse events, only local adverse events, only systemic adverse events, or both. **(c)** Adverse events perceived by individuals were ranked and classified by severity (none, low, moderate, high). Comparisons between groups were analysed using the X^2^ test.

Vaccine-related adverse events were compared between patients and controls in the first week after both vaccinations based on self-reporting using a questionnaire. Overall, both vaccinations were well tolerated with pain at the injection site followed by redness at the injection site and fatigue being most frequently reported (Fig. 7a). Compared with healthy controls, patients on dialysis reported fewer local and/or systemic adverse events (Fig. 7b). In general, most controls reported similarly frequent adverse events after the first and the second vaccination. In contrast, adverse events among patients tended to be less frequent after the second vaccination, with the exception of fatigue and headache (Fig. 7c).

## Discussion

The recombinant vaccine HZ/su was approved for the prevention of herpes zoster and associated serious complications in immunocompromised patients such as patients on dialysis, who are at increased risk of VZV reactivation due to uremic immunodeficiency.^9^^,^^10^ Knowledge on the immunogenicity and reactogenicity after vaccination with HZ/su in patients on dialysis in relation to immunocompetent controls was limited. In this observational study, we show that two standard doses of the HZ/su vaccine were well tolerated and induced a multifunctional VZV-specific CD4 T-cell response in patients on dialysis. In contrast, CD8 T-cells were only poorly induced. At the humoral level, we also observed an increase in VZV-specific IgG antibodies and neutralizing activity. Overall, both cellular and humoral immune responses were slightly less pronounced in patients than in immunocompetent controls, and long-term levels of VZV-specific CD4 T-cells and IgG after one year were less durable.

Our baseline results are consistent with previous observations of similar steady state levels of VZV-specific cellular and humoral immunity in patients on dialysis and controls,^33^ and with previous observations after VZV-vaccination of healthy controls.^4^^,^^23^^,^^34^, ^35^, ^36^ We now show that the HZ/su vaccine induced a significant increase in VZV-specific CD4 T-cell levels in patients on dialysis. We have previously shown that VZV reactivation in patients with herpes zoster or meningitis leads to an upregulation of CTLA-4 on VZV-specific T-cells, which normalized after resolution of symptoms.^30^^,^^31^ Similar dynamics of CTLA-4 expression was now also observed for vaccine-induced T-cells and thereafter. This suggests that CTLA-4 expression on VZV-specific CD4 T-cells may represent a direct measure for recent antigen encounter resulting from either infection or vaccination. In contrast, the cytokine profile seems to differ during infection and vaccination, as vaccine-induced VZV-specific CD4 T-cells showed a multifunctional Th1 phenotype, whereas Th1 CD4 T-cells after acute zoster were functionally restricted and predominantly expressed IFNγ only.^31^ In general, the quantitative and functional increase in vaccine-specific CD4 T-cells after the second vaccination may contribute to better protection through proliferation and secretion of effector molecules^37^ and emphasizes the importance of the booster dose to establish a robust immune response. However, compared to controls, patients on dialysis mounted significantly lower VZV-specific CD4 T-cell levels after both vaccinations which remained lower at one year follow-up. Phenotypical analysis of vaccine-induced CD4 memory cells based on CD27 and CD45RO expression revealed a higher proportion of central memory T-cells and lower proportion of effector memory T-cells in patients compared to controls. Similar observations of an impaired effector memory CD4 T-cell response were made in patients on dialysis two weeks after hepatitis B vaccination.^38^ This may result from the fact that patients on dialysis have a higher apoptosis rate of T-cells.^39^^,^^40^ As central memory T-cells are better protected against apoptosis,^41^^,^^42^ apoptosis may preferentially affect effector memory T-cells in patients. It is unlikely that lower levels of vaccine-induced T cells or antibodies are directly related to the effects of the dialysis procedure itself, as blood samples were always drawn prior to a dialysis session (i.e. 44–68 h after the last), and neither antibody levels nor lymphocyte populations^43^ are overtly affected due to a pore size of approximately 15–20 kDa and due to increasing biocompatibility of dialysis membranes.

Compared to the VZV-specific CD4 T-cell levels, specific CD8 T-cells were poorly induced in both patients on dialysis and controls, and were primarily detected after proliferation upon longer stimulation times. This is consistent with other studies on the HZ/su vaccine,^35^^,^^44^ and is a typical feature of protein-based vaccines in general, which are predominantly presented via the MHC class II pathway and thus primarily activate CD4 T-cells. This is also illustrated by the protein-based SARS-CoV-2 vaccine NVX-CoV2373, where vaccine-induced CD8 T-cell levels were significantly lower as compared to vector-based or mRNA vaccines.^28^^,^^45^^,^^46^ Based on the observation that the HZ/su vaccine is highly effective,^22^^,^^23^ CD4 T-cells may have a dominant role in mediating protection from herpes zoster.^47^ Interestingly, despite low levels of VZV-specific CD8 T-cells after short-term stimulation ex vivo, proliferation of specific CD8 T-cells became detectable after 7 days, albeit to a lesser extent than CD4 T-cells. It is possible that protein-derived peptides are presented to MHC class I via cross-presentation and recognized by CD8 T-cells. The AS01_B_ adjuvant used for HZ/su contains MLP/QS21, which is known to improve antigen cross-presentation^48^ may thereby induce CD8 T-cells and also increase their cytotoxic activity.^49^

Although the induction of the humoral VZV response appears to play a less important role in preventing VZV reactivation,^50^^,^^51^ it may have a role in neutralizing the virus and/or preventing symptomatic disease during reexposure.^5^^,^^52^ In line with a high VZV-seroprevalence in the world population,^53^ the majority of individuals in our study were already seropositive before the first vaccination. As chickenpox vaccination is only recommended in Germany since 2004,^54^ most individuals can be considered to have experienced a natural primary VZV infection earlier in life. Only two patients and four controls had anti-VZV-gE antibodies below detection limit prior to vaccination. However, as these individuals reported a history of chickenpox, their antibody levels may already have decreased below the detection limit by the time of analysis. In our study, the HZ/su vaccine led to a further induction of VZV-specific IgG with strong neutralization activity in both patients and controls. However, as with CD4 T-cells, the increase in specific IgG were less pronounced in patients on dialysis and were less stable over time. In our study, we characterized the circulating fraction of Tfh-cells in the blood as surrogate population of Tfh-cells that interact with B-cells in secondary lymphoid organs and provide support in the production of high-affinity antibodies. However, we were unable to reveal pronounced vaccination-induced changes, which may be due to the fact that Tfh-cell levels were identified in an antigen-non-specific manner, and that results from circulating Tfh-cells may not reflect dynamic changes in the lymph nodes. Likewise, no vaccine-induced changes in plasmablasts of patients on dialysis and controls were detectable in circulation two weeks after each vaccination. This may be due to the fact that peak levels of plasmablasts are generally observed earlier within the first week after vaccinations^55^ or after herpes zoster reactivation^56^ with a subsequent decrease in levels. In our study, patients on dialysis generally showed significantly lower numbers of B-cells with an imbalance in B-cell subpopulations such as higher levels of naive B-cells and lower levels of plasmablasts and switched memory B-cells, which may contribute to the state of uremic immunodeficiency.^57^^,^^58^ Similar differences in the distribution of B-cell subpopulations have also been described in other diseases such as systemic sclerosis.^59^ One reason for the higher percentage of naive B-cells may be increased production to maintain homeostasis, while a lower percentage of non-switched B-cells in patients is possibly caused by increased apoptosis.^60^

In our study, we show that the HZ/su vaccine was well tolerated by patients on dialysis, which is in line with a recent adherence and safety study.^61^ Local adverse events were more frequently reported as compared to systemic reactions, and overall less frequent than in controls, which has already been observed with vaccinations against SARS-CoV-2.^62^ This may be explained by the fact that patients on dialysis often suffer from chronic complaints such as fatigue or headaches due to their uremic disease and the dialysis procedure,^63^ so that vaccination reactions, especially systemic ones, are perceived as less severe. Thus, both the good immunogenicity as well as the low reactogenicity of the vaccine may increase compliance to adherence with vaccine recommendations and thus contribute to a reduction in cases of herpes zoster and postherpetic neuralgia.

The strength of our study is the investigation of both humoral and cellular immunity including phenotypical characterization of VZV-specific CD4 T-cells in direct comparison with immunocompetent controls, which to our knowledge has not been investigated before. Similar results have been reported in transplant recipients,^25^, ^26^, ^27^ although no control group was included to specifically analyse the effect of immunodeficiency. Limitations include the low overall number of individuals and lack of clinical follow-up beyond one year, which does not provide information on effectiveness. Nevertheless, sample size was sufficient to characterize immunogenicity and reactogenicity, and reveal differences between controls and patients. As the number of blood samples was limited for ethical and logistical reasons, time points for sampling was optimized for detection of antigen-specific antibodies and T-cells, whereas analysis of plasmablasts would have required earlier analysis.^55^ Likewise, the number of markers to characterization of T-cell subpopulations was limited and did not include CCR7 or CD62L which are more commonly used as markers for differentiation and homing.^64^ Unlike after SARS-CoV-2 vaccination, HZ/su induced a specific CD4 T-cell response and IgG in the majority of patients after two doses of vaccine.^65^^,^^66^ It is very likely that patients on dialysis benefit from the previous contact with VZV during natural infection and thus from a pre-existing immune response that was boosted by the two HZ/su vaccine doses. In a study with healthy individuals, robust immunogenicity of HZ/su has already been shown to persist over a period of 10 years.^67^ Similar long-term follow-up data are not yet available for immunocompromised individuals but are important in light of our findings that cellular and humoral immunity appears less stable in patients on dialysis than in controls. A more rapid loss of vaccine-induced immune responses has already been described after influenza vaccination of patients on dialysis, while the immune response in healthy individuals remained more stable.^15^ While yearly influenza vaccination is recommended, future studies should clarify whether patients on dialysis may benefit from additional doses of HZ/su vaccines. On an individual level, future studies should determine thresholds for cellular and/or humoral immune response parameters associated with increasing risk of VZV reactivation.

In conclusion, we showed that the inactivated herpes zoster HZ/su vaccine was well tolerated and induced specific antibodies and polyfunctional CD4 T-cells in both patients and controls. Quantitative and qualitative differences in cellular and humoral immunity between patients and controls may indicate reduced duration of protection. Future observational studies, including efficacy data under real-world conditions, should indicate whether further booster doses may be required in dialysis-patients.

## Contributors

F.H., T.S., D.S., M.E., U.S., and M.S. designed the study and the experiments, F.H. and D.S performed experiments; F.H., S.L., M.G., K. B., J.M., U.S., and M.S. contributed to study design, patient recruitment, and clinical data acquisition. F.H. and M.S. have verified the underlying data, and have performed statistical analysis. T.S., D.S., and M.S. supervised all parts of the study; F.H., and M.S. wrote the manuscript. All authors read and approved the final version of the manuscript.

## Data sharing statement

Data related to this study are available within the text, figures, and tables. All are available from the corresponding author upon request (martina.sester@uks.eu).

## Declaration of interests

M.S. has received grant support from Astellas and Biotest to the organization Saarland University outside the submitted work, and honoraria for lectures from Biotest and Novartis, and for advisory boards from Moderna, Biotest, MSD and Takeda outside the submitted work. T.S. has received travel grant support from Biotest outside the submitted work. All other authors of this manuscript have no conflicts of interest to disclose.

## References

[1] Hope-Simpson R.E. (1965). The nature of herpes zoster: a long-term study and a new hypothesis. Proc Roy Soc Med.

[2] Mueller N.H., Gilden D.H., Cohrs R.J., Mahalingam R., Nagel M.A. (2008). Varicella zoster virus infection: clinical features, molecular pathogenesis of disease, and latency. Neurol Clin.

[3] Schmader K.E., Sloane R., Pieper C. (2007). The impact of acute herpes zoster pain and discomfort on functional status and quality of life in older adults. Clin J Pain.

[4] Levin M.J., Smith J.G., Kaufhold R.M. (2003). Decline in varicella-zoster virus (VZV)-specific cell-mediated immunity with increasing age and boosting with a high-dose VZV vaccine. J Infect Dis.

[5] Arvin A.M. (2008). Humoral and cellular immunity to varicella-zoster virus: an overview. J Infect Dis.

[6] Yawn B.P., Gilden D. (2013). The global epidemiology of herpes zoster. Neurology.

[7] Chen S.Y., Suaya J.A., Li Q. (2014). Incidence of herpes zoster in patients with altered immune function. Infection.

[8] (2018). Ständige Impfkommission (STIKO): Wissenschaftliche Begründung zur Empfehlung einer Impfung mit dem Herpes zoster-subunit-Totimpfstoff. Epid Bull.

[9] Kuo C.C., Lee C.T., Lee I.M., Ho S.C., Yang C.Y. (2012). Risk of herpes zoster in patients treated with long-term hemodialysis: a matched cohort study. Am J Kidney Dis.

[10] Li Z., Wang Q., Ma J. (2021). Risk factors for herpes zoster in patients with chronic Kidney disease: a case-control study. Vaccines.

[11] Girndt M., Sester M., Sester U., Kaul H., Köhler H. (2001). Molecular aspects of T–and B-cell function in uremia. Kidney Int.

[12] Sarnak M.J., Jaber B.L. (2000). Mortality caused by sepsis in patients with end-stage renal disease compared with the general population. Kidney Int.

[13] Girndt M., Pietsch M., Köhler H. (1995). Tetanus immunization and its association to hepatitis B vaccination in patients with chronic renal failure. Am J Kidney Dis.

[14] Kreft B., Klouche M., Kreft R., Kirchner H., Sack K. (1997). Low efficiency of active immunization against diphtheria in chronic hemodialysis patients. Kidney Int.

[15] Sester U., Schmidt T., Kuhlmann M.K., Gärtner B.C., Uhlmann-Schiffler H., Sester M. (2013). Serial influenza-vaccination reveals impaired maintenance of specific T-cell memory in patients with end-stage renal failure. Vaccine.

[16] Rautenberg P., Proppe D., Schütte A., Ullmann U. (1989). Influenza subtype-specific immunoglobulin A and G responses after booster versus one double-dose vaccination in hemodialysis patients. Eur J Clin Microbiol Infect Dis.

[17] Siedler A., Koch J., Garbe E. (2019). Background paper to the decision to recommend the vaccination with the inactivated herpes zoster subunit vaccine. Bundesgesundheitsblatt Gesundheitsforschung Gesundheitsschutz.

[18] Robert-Koch-Institut R (2018). Wissenschaftliche Begründung zur Empfehlung einer Impfung mit dem Herpes zoster-subunit-Totimpfstoff. Epidemiol Bull.

[19] Robert Koch-Institut R (2017). Epidemiologisches Bulletin 36/2017 Empfehlungen zur Impfung gegen Herpes Zoste.

[20] Mwakingwe-Omari A., Lecrenier N., Naficy A., Curran D., Posiuniene I. (2023). Recombinant zoster vaccine in immunocompetent and immunocompromised adults: a review of clinical studies. Hum Vaccin Immunother.

[21] Lecrenier N., Beukelaers P., Colindres R. (2018). Development of adjuvanted recombinant zoster vaccine and its implications for shingles prevention. Expet Rev Vaccine.

[22] Cunningham A.L., Lal H., Kovac M. (2016). Efficacy of the herpes zoster subunit vaccine in adults 70 Years of age or older. N Engl J Med.

[23] Lal H., Cunningham A.L., Godeaux O. (2015). Efficacy of an adjuvanted herpes zoster subunit vaccine in older adults. N Engl J Med.

[24] Bastidas A., de la Serna J., El Idrissi M. (2019). Effect of recombinant zoster vaccine on incidence of herpes zoster after autologous stem cell transplantation: a randomized clinical trial. JAMA.

[25] Vink P., Ramon Torrell J.M., Sanchez Fructuoso A. (2020). Immunogenicity and safety of the adjuvanted recombinant zoster vaccine in chronically immunosuppressed adults following renal transplant: a phase 3, randomized clinical trial. Clin Infect Dis.

[26] L'Huillier A.G., Hirzel C., Ferreira V.H. (2021). Evaluation of recombinant herpes zoster vaccine for primary immunization of varicella-seronegative transplant recipients. Transplantation.

[27] Hirzel C., L'Huillier A.G., Ferreira V.H. (2021). Safety and immunogenicity of adjuvanted recombinant subunit herpes zoster vaccine in lung transplant recipients. Am J Transplant.

[28] Hielscher F., Schmidt T., Klemis V. (2022). NVX-CoV2373-induced cellular and humoral immunity towards parental SARS-CoV-2 and VOCs compared to BNT162b2 and mRNA-1273-regimens. J Clin Virol.

[29] Schoch J., Rohrer T.R., Kaestner M. (2017). Quantitative, phenotypical, and functional characterization of cellular immunity in children and adolescents with down syndrome. J Infect Dis.

[30] Schub D., Fousse M., Faßbender K. (2018). CTLA-4-expression on VZV-specific T cells in CSF and blood is specifically increased in patients with VZV related central nervous system infections. Eur J Immunol.

[31] Schub D., Janssen E., Leyking S. (2015). Altered phenotype and functionality of varicella zoster virus-specific cellular immunity in individuals with active infection. J Infect Dis.

[32] Sester U., Presser D., Dirks J., Gärtner B.C., Köhler H., Sester M. (2008). PD-1 expression and IL-2 loss of cytomegalovirus- specific T cells correlates with viremia and reversible functional anergy. Am J Transplant.

[33] Rondaan C., de Joode A.A.E., van Assen S., Bos N.A., Westerhuis R., Westra J. (2018). Increased incidence of herpes zoster in patients on renal replacement therapy cannot be explained by intrinsic defects of cellular or humoral immunity to varicella-zoster virus. Antivir Res.

[34] Chlibek R., Pauksens K., Rombo L. (2016). Long-term immunogenicity and safety of an investigational herpes zoster subunit vaccine in older adults. Vaccine.

[35] Leroux-Roels I., Leroux-Roels G., Clement F. (2012). A phase 1/2 clinical trial evaluating safety and immunogenicity of a varicella zoster glycoprotein E subunit vaccine candidate in young and older adults. J Infect Dis.

[36] Cunningham A.L., Heineman T.C., Lal H. (2018). Immune responses to a recombinant glycoprotein E herpes zoster vaccine in adults aged 50 years or older. J Infect Dis.

[37] Darrah P.A., Patel D.T., De Luca P.M. (2007). Multifunctional TH1 cells define a correlate of vaccine-mediated protection against Leishmania major. Nat Med.

[38] Litjens N.H., Huisman M., van den Dorpel M., Betjes M.G. (2008). Impaired immune responses and antigen-specific memory CD4+ T cells in hemodialysis patients. J Am Soc Nephrol.

[39] Moser B., Roth G., Brunner M. (2003). Aberrant T cell activation and heightened apoptotic turnover in end-stage renal failure patients: a comparative evaluation between non-dialysis, haemodialysis, and peritoneal dialysis. Biochem Biophys Res Commun.

[40] Meier P., Dayer E., Blanc E., Wauters J.-P. (2002). Early T cell activation correlates with expression of apoptosis markers in patients with end-stage renal disease. J Am Soc Nephrol.

[41] Mahnke Y.D., Brodie T.M., Sallusto F., Roederer M., Lugli E. (2013). The who's who of T-cell differentiation: human memory T-cell subsets. Eur J Immunol.

[42] Riou C., Yassine-Diab B., Van grevenynghe J. (2007). Convergence of TCR and cytokine signaling leads to FOXO3a phosphorylation and drives the survival of CD4+ central memory T cells. J Exp Med.

[43] Sester U., Sester M., Heine G., Kaul H., Girndt M., Kohler H. (2001). Strong depletion of CD14(+)CD16(+) monocytes during haemodialysis treatment. Nephrol Dial Transplant.

[44] Dendouga N., Fochesato M., Lockman L., Mossman S., Giannini S.L. (2012). Cell-mediated immune responses to a varicella-zoster virus glycoprotein E vaccine using both a TLR agonist and QS21 in mice. Vaccine.

[45] Zhang Z., Mateus J., Coelho C.H. (2022). Humoral and cellular immune memory to four COVID-19 vaccines. Cell.

[46] Klemis V., Schmidt T., Schub D. (2022). Comparative immunogenicity and reactogenicity of heterologous ChAdOx1-nCoV-19-priming and BNT162b2 or mRNA-1273-boosting with homologous COVID-19 vaccine regimens. Nat Commun.

[47] Chlibek R., Smetana J., Pauksens K. (2014). Safety and immunogenicity of three different formulations of an adjuvanted varicella-zoster virus subunit candidate vaccine in older adults: a phase II, randomized, controlled study. Vaccine.

[48] den Brok M.H., Büll C., Wassink M. (2016). Saponin-based adjuvants induce cross-presentation in dendritic cells by intracellular lipid body formation. Nat Commun.

[49] Mikloska Z., Rückholdt M., Ghadiminejad I., Dunckley H., Denis M., Cunningham A.L. (2000). Monophosphoryl lipid A and QS21 increase CD8 T lymphocyte cytotoxicity to herpes simplex virus-2 infected cell proteins 4 and 27 through IFN-γ and IL-12 production 1. J Immunol.

[50] Burke B.L., Steele R.W., Beard O.W., Wood J.S., Cain T.D., Marmer D.J. (1982). Immune responses to varicella-zoster in the aged. Arch Intern Med.

[51] Webster A., Grint P., Brenner M.K., Prentice H.G., Griffiths P.D. (1989). Titration of IgG antibodies against varicella zoster virus before bone marrow transplantation is not predictive of future zoster. J Med Virol.

[52] Arvin A.M., Koropchak C.M., Wittek A.E. (1983). Immunologic evidence of reinfection with varicella-zoster virus. J Infect Dis.

[53] Kilgore P.E., Kruszon-Moran D., Seward J.F. (2003). Varicella in Americans from NHANES III: implications for control through routine immunization. J Med Virol.

[54] Robert Koch Institut (2004). https://www.rki.de/DE/Content/Infekt/EpidBull/Archiv/2004/Ausgabenlinks/49_04.pdf?__blob=publicationFile.

[55] Fink K. (2012). Origin and function of circulating plasmablasts during acute viral infections. Front Immunol.

[56] Fukuchi K., Shimauchi T., Tatsuno K., Tokura Y. (2018). Induction of plasmablasts by follicular helper T cell-CXCL13 axis upon occurrence of herpes zoster. Clin Immunol.

[57] Molina M., Allende L.M., Ramos L.E. (2018). CD19(+) B-cells, a new biomarker of mortality in hemodialysis patients. Front Immunol.

[58] Descamps-Latscha B., Chatenoud L. (1996). T cells and B cells in chronic renal failure. Semin Nephrol.

[59] Simon D., Balogh P., Bognár A. (2016). Reduced non-switched memory B cell subsets cause imbalance in B cell repertoire in systemic sclerosis. Clin Exp Rheumatol.

[60] Fernández-Fresnedo G., Ramos M.A., González-Pardo M.C., de Francisco A.L., López-Hoyos M., Arias M. (2000). B lymphopenia in uremia is related to an accelerated in vitro apoptosis and dysregulation of Bcl-2. Nephrol Dial Transplant.

[61] Martino F.K., Pini S., Scaparrotta G. (2023). Recombinant Varicella Zoster vaccine in haemodialysis facilities: adherence and safety. J Nephrol.

[62] Bronder S., Mihm J., Urschel R. (2024). Potent induction of humoral and cellular immunity after bivalent BA.4/5 mRNA vaccination in dialysis patients. NPJ Vaccines.

[63] Caplin B., Kumar S., Davenport A. (2011). Patients' perspective of haemodialysis-associated symptoms. Nephrol Dial Transplant.

[64] Sallusto F., Geginat J., Lanzavecchia A. (2004). Central memory and effector memory T cell subsets: function, generation, and maintenance. Annu Rev Immunol.

[65] Simon B., Rubey H., Treipl A. (2021). Haemodialysis patients show a highly diminished antibody response after COVID-19 mRNA vaccination compared with healthy controls. Nephrol Dial Transplant.

[66] Espi M., Charmetant X., Barba T. (2021). The ROMANOV study found impaired humoral and cellular immune responses to SARS-CoV-2 mRNA vaccine in virus-unexposed patients receiving maintenance hemodialysis. Kidney Int.

[67] Strezova A., Diez-Domingo J., Al Shawafi K. (2022). Long-term protection against herpes zoster by the adjuvanted recombinant zoster vaccine: Interim efficacy, immunogenicity, and safety results up to 10 years after initial vaccination. Open Forum Infect Dis.

