# Supplementary material for: The inactivated herpes zoster vaccine HZ/su induces a varicella zoster virus specific cellular and humoral immune response in patients on dialysis
Source: eBioMedicine. 2024 Sep 11;108:105335. doi: 10.1016/j.ebiom.2024.105335 (PMC11416227; doi:10.1016/j.ebiom.2024.105335)
Supplement: Supplementary Figures and Tables [file mmc1.docx]

# Supplementary information

# The inactivated herpes zoster vaccine HZ/su induces a varicella zoster virus specific cellular and humoral immune response in patients on dialysis

Running head: VZV-specific immunity in patients on dialysis

Franziska Hielscher, MSc, Tina Schmidt, PhD, Martin Enders, MD, Sarah Leyking, MD, Markus Gerhart, MD, Kai van Bentum, MD, Janine Mihm, MD, David Schub, PhD, Urban Sester, MD, and Martina Sester, PhD

This supplement contains

- Supplementary table S1-S3
- Supplementary figures S1-S5.

# Supplementary tables

## Table S1: Underlying diseases in patients on dialysis

| **Underlying disease** | **n (%)** |
| --- | --- |
| Chronic glomerulonephritis | 4 (13.8) |
| Hereditary nephropathy | 4 (13.8)^$^ |
| Urological nephropathies (obstructive, nephrectomy) | 4 (13.8) |
| Diabetic nephropathy | 6 (20.7) |
| Hypertensive/vascular nephropathy | 6 (20.7) |
| Others | 5 (17.2)^#^ |

^$^incl. autosomal dominant polycystic kidney disease; ^#^incl. lipidapheresis (n=1).

## Table S2: Antibodies used for flow-cytometric analyses

| **Antigen** | **Conjugate** | **Clone** | **Isotype** | **Reactivity** | **Catalogue number, RRID^1^** |
| --- | --- | --- | --- | --- | --- |
| CD3 | PerCP | SK7 | IgG1, κ | mouse anti-human | Cat #345766  RRID: AB_2783791 |
| CD3 | APC | SK7 | IgG1, κ | mouse anti-human | Cat #345767  RRID:AB_2833003 |
| CD4 | APC-H7 | SK3 | IgG1, κ | mouse anti-human | Cat #641398  RRID: AB_1645732 |
| CD4 | PE | SK3 | IgG1, κ | mouse anti-human | Cat #345769  RRID:AB_2728699 |
| CD8 | PerCP | SK1 | IgG1, κ | mouse anti-human | Cat #345774  RRID: AB_2868802 |
| CD8 | V500 | RPA-T8 | IgG1, κ | mouse anti-human | Cat #560774  RRID: AB_1937325 |
| CD19 | FITC | HIB19 | IgG1, κ | mouse anti-human | Cat #555412  RRID: AB_395812 |
| CD27 | APC | L128 | IgG1 | mouse anti-human | Cat #337169  RRID: AB_647368 |
| CD27 | FITC | L128 | IgG1, κ | mouse anti-human | Cat #340424  RRID:AB_400031 |
| CD38 | PE | HB7 | IgG1, κ | mouse anti-human | Cat #345806  RRID: AB_2868828 |
| CD45RO | PE-Cy7 | UCHL-1 | IgG2a, κ | mouse anti-human | Cat #337168  RRID:AB_647426 |
| CD69 | PE-Cy7 | L78 | IgG1, κ | mouse anti-human | Cat #335792,  RID: AB_1937286 |
| CD69 | PerCP | L78 | IgG1, κ | mouse anti-human | Cat #340548  RRID:AB_400054 |
| CTLA-4 | APC | BNI3 | IgG2a, κ | mouse anti-human | Cat #555855  RRID: AB_398615 |
| CXCR5 | PerCP-Cy5.5 | RF8B2 | IgG2b k | rat anti-human | Cat #562781  RRID:AB_2313576 |
| ICOS | PE | DX29 | IgG1, κ | mouse anti-human | Cat #557802  RRID:AB_396878 |
| IFNγ | FITC | 4S·B3 | IgG1, κ | mouse anti-human | Cat #554551  RRID: AB_395473 |
| IgD | PE-Cy7 | IA6-2 | IgG2a, κ | mouse anti-human | Cat #561314  RRID: AB_10642457 |
| IL-2 | PE | MQ1-17H12 | IgG2a, κ | rat anti-human | Cat #559334  RRID: AB_397231 |
| PD-1 | APC | MIH4 | IgG1, κ | mouse anti-human | Cat #558694  RRID:AB_1645458 |
| TNFα | V450 | MAb11 | IgG1, κ | mouse anti-human | Cat #561311  RRID: AB_10646031 |

^1^RRID, Research Resource ID; all antibodies were separately titrated in house to evaluate best concentrations for discrimination of negative and positive cell populations. Finally, panels including defined concentration of antibodies were tested together to confirm that identified antibody concentrations are also sufficient when compensations are required; all antibodies from BD Biosciences, Heidelberg, Germany.

## Table S3: Mixed-effects analysis of parameters of patients compared to controls over time

|  | total (time)* | pre v1 | post v1 | pre v2 | post v2 | Follow Up |
| --- | --- | --- | --- | --- | --- | --- |
| % VZV-specific CD4 T-cells | **<0.0001** | 0.0586 | 0.4115 | 0.8598 | **0.0499** | 0.392 |
| % SEB-reactive CD4 T-cells | 0.4169 | **0.0009** | **0.0021** | **0.0039** | **0.0071** | **0.0065** |
| % VZV-specific CD8 T-cells | **0.0281** | 0.0707 | 0.871 | 0.9442 | 0.6373 | 0.9867 |
| % SEB-reactive CD8 T-cells | 0.5186 | **0.0026** | **0.0066** | **0.0019** | **0.002** | **0.0236** |
| CTLA-4 MFI VZV-specific CD4 T-cells | **0.0149** | n.a. | **0.0028** | n.a. | **0.0037** | **0.0163** |
| CTLA-4 MFI SEB-reactive CD4 T-cells | **0.0142** | 0.9033 | 0.6288 | 0.3613 | 0.3601 | 0.8971 |
| % proliferated VZV-specific CD4 T-cells | **<0.0001** | 0.444 | 0.1222 | 0.7077 | 0.4094 | 0.4994 |
| % proliferated SEB-reactive CD4 T-cells | 0.0673 | 0.2428 | 0.5995 | **0.0155** | 0.7421 | 0.9645 |
| % proliferated VZV-specific CD8 T-cells | **<0.0001** | 0.9912 | 0.2215 | 0.0775 | 0.3669 | 0.073 |
| % proliferated SEB-reactive CD8 T-cells | 0.374 | 0.3972 | 0.7664 | 0.2366 | 0.6626 | 0.931 |
| % plasmablasts | 0.469 | 0.364 | 0.2099 | 0.1444 | 0.2193 | 0.3241 |
| % follicular T-helper cells | **0.0038** | **0.0034** | 0.2314 | 0.1507 | 0.0895 | 0.2754 |
| VZV-specific IgGs (IU/l) | **<0.0001** | 0.4883 | 0.5281 | 0.5372 | **0.0326** | 0.0635 |
| Neutralizing titer (GMT) | **<0.0001** | 0.0963 | n.a. | n.a. | 0.0715 | 0.1299 |

Data are derived from figure 1, figure 2, figure 4, figure 5, figure 6, and supplementary figure S4; analyses were performed using two-way ANOVA (mixed-effects analysis with Tukey´s post test), significant p-values are marked in bold. *refers to overall differences between patients and controls.

# Supplementary figures

## Figure S1


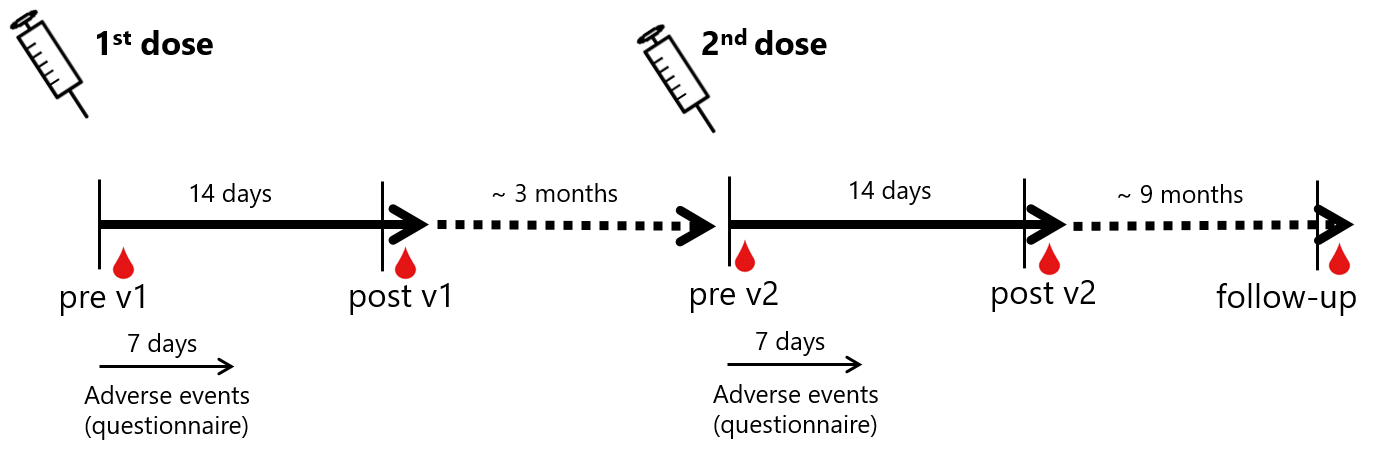


**Figure S1: Schematic representation of study design.** Patients and controls received two doses of the Hz/su vaccine, and blood samples were drawn before each vaccination as well as two weeks after the first and the second vaccination. In addition, a final blood sample was drawn 12 months after the first vaccination (i.e. 9 months after the second). Adverse events were self-reported within the first week after the first and the second vaccination using a standardized questionnaire.

## Figure S2


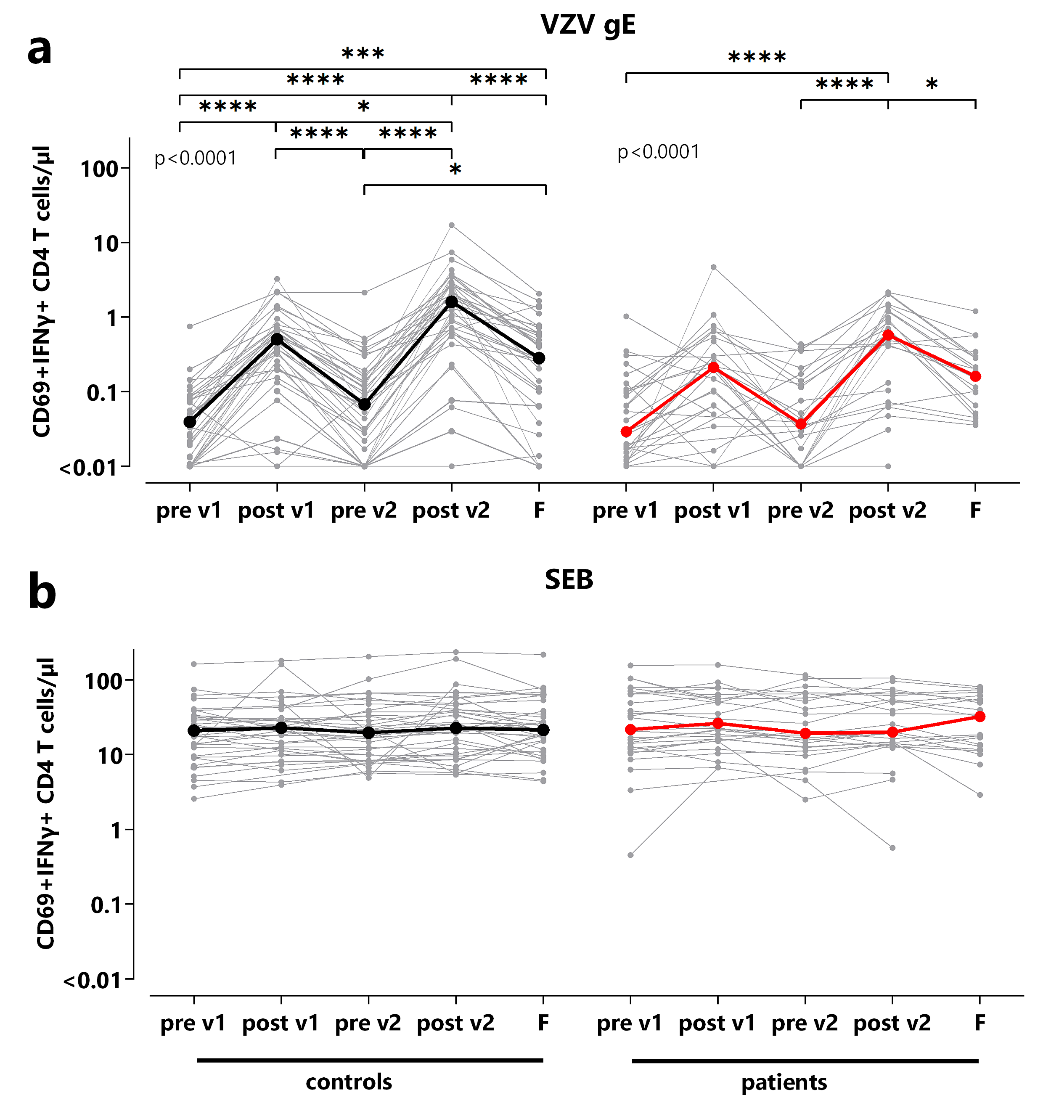


**Figure S2: Characterization of absolute numbers of VZV-specific CD4 T cells over time.** The absolute numbers of **(a)** VZV-specific and **(b)** SEB-reactive CD4 T cells were calculated from the percentage of CD69+ IFNγ+ CD4 T cells and the corresponding differential blood counts. Data for controls (black) and patients (red) and shown over time. The bold lines represent medians. Statistical analysis was carried out using the Friedman test with Dunn post-test. VZV, varizella zoster virus, SEB, *Staphylococcus aureus* enterotoxin B.

## Figure S3


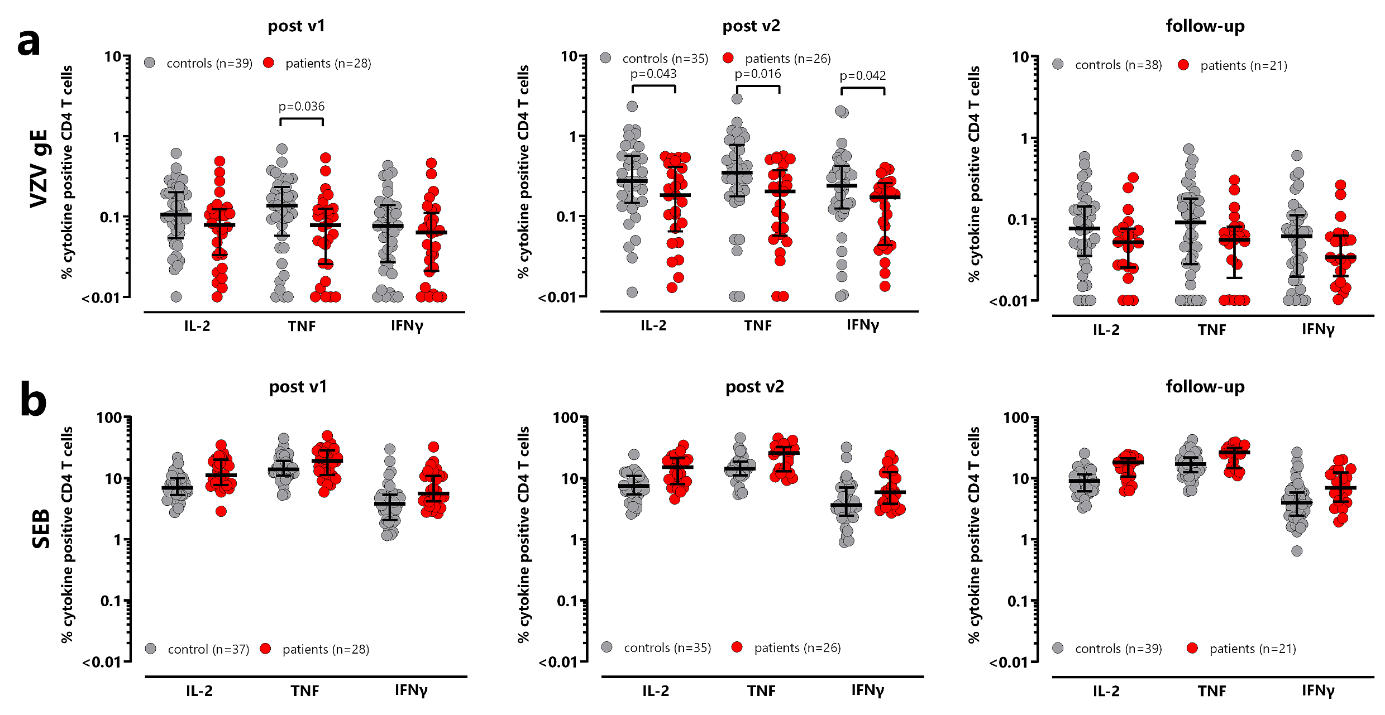


**Figure S3: Cytokine expression of VZV-specific and SEB-reactive CD4 T-cells. (a)** VZV-specific or **(b)** SEB-reactive CD4 T-cells were stimulated and CD69-positive CD4 T-cells producing IL-2, TNF or IFNγ were quantified after the first, the second vaccination and on follow-up one year after the first vaccination. VZV-specific CD4 T-cell levels are displayed after subtraction of negative control values. Samples from healthy controls (red) and patients on dialysis (grey) were compared at each time point. Bars represent median values with interquartile ranges. Statistical analysis was performed using Mann-Whitney test. IL-2, interleukin 2; IFN, interferon; TNF, tumor necrosis factor, VZV, *Varicella zoster virus*; SEB, *Staphylococcus aureus* enterotoxin B.

## Figure S4

**
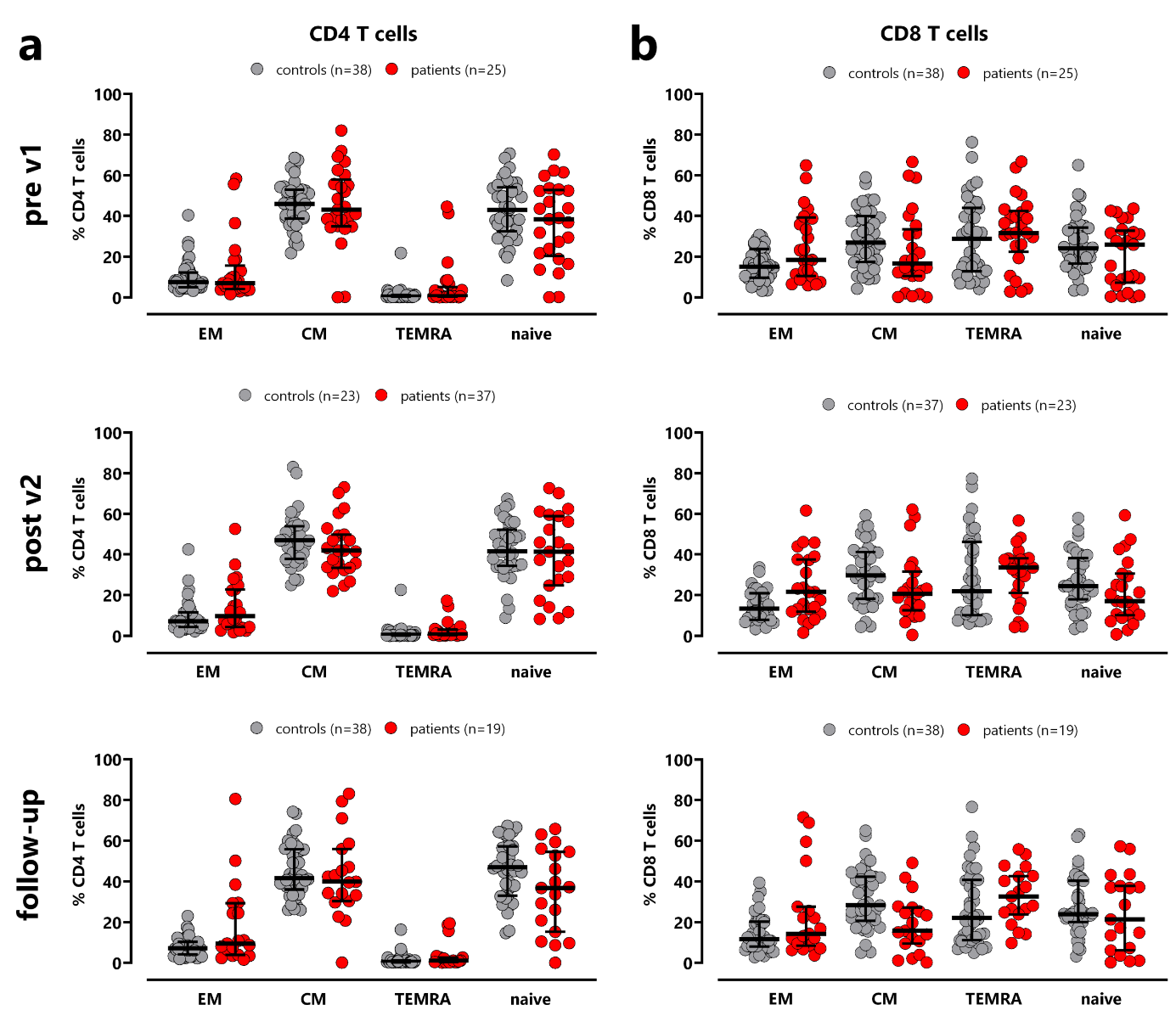
**

**Figure S4: CD4 and CD8 T-cell differentiation in patients on dialysis** **and controls.** Differentiation status of bulk **(a)** CD4 T-cells or **(b)** CD8 T-cells classified into naive, central memory (CM), effector memory (EM), and terminally differentiated effector memory (TEMRA) cells based on expression of CD45RO and CD27. T-cell populations were compared between controls (gray) and patients on dialysis (red) after the first and the second vaccinations and on follow-up. Statistical analysis was performed using Mann-Whitney test. Bars represent medians with interquartile ranges.

## Figure S5


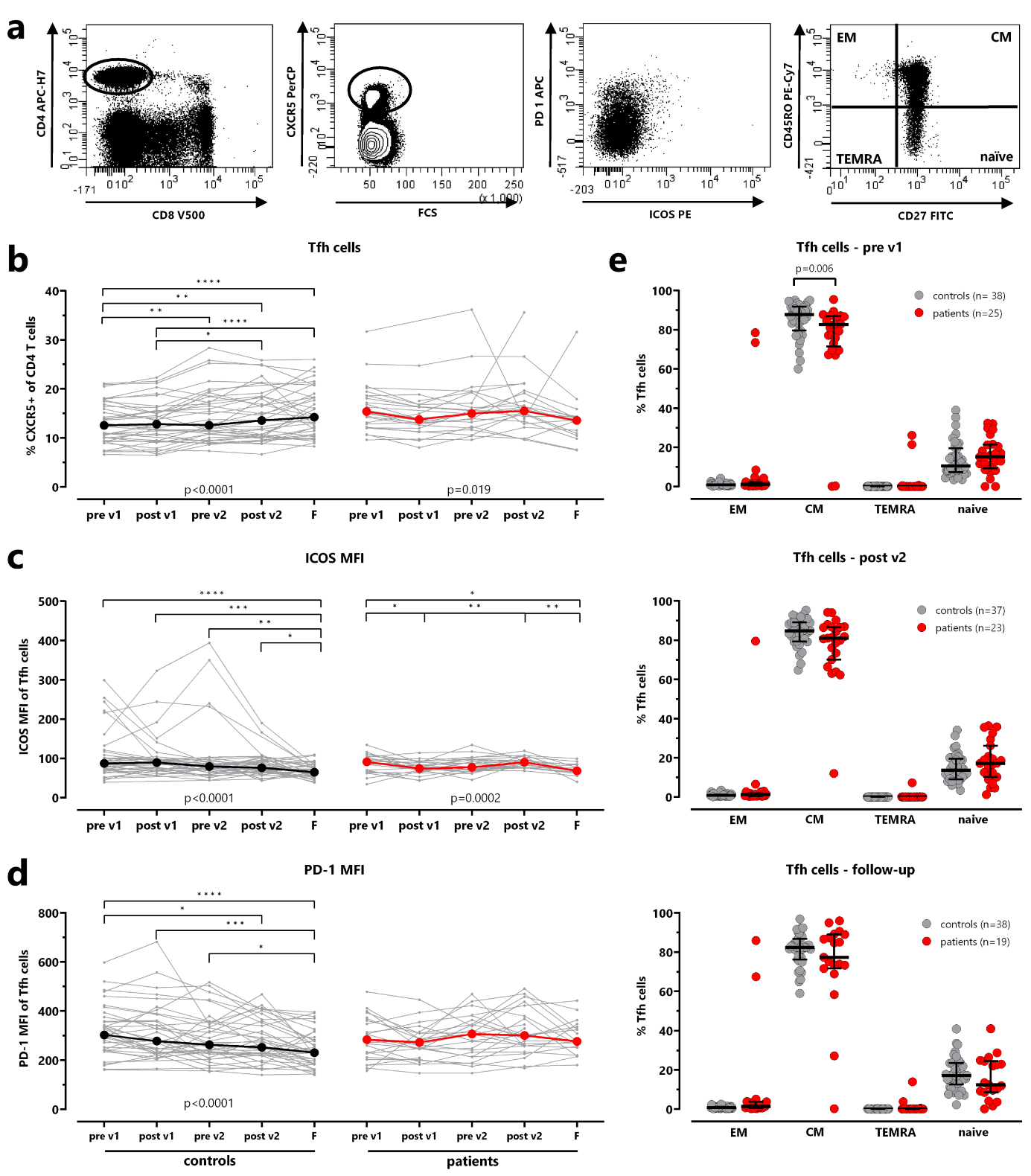


**Figure S5: Characterization of follicular T helper cells. (a)** Representative plots of follicular T helper (Tfh) cells identified as CXCR5+ CD4 T-cells. Tfh-cells were analysed for expression of PD-1 and ICOS, and the differentiation into naive, central memory (CM), effector memory (EM), and terminally differentiated effector memory (TEMRA) was determined using CD45RO and CD27. **(b)** The percentage of Tfh-cells among CD4 T-cells, and the median fluorescence intensity (MFI) of **(c)** ICOS and **(d)** PD-1 were determined over time in controls (black) and patients on dialysis (red). Bold lines represent medians and Friedman test was performed for paired analyses. **(e)** The differentiation status of Tfh-cells before the first vaccination, after the second vaccination and after one year (follow-up) is shown in controls (grey) and patients (red). Bars represent medians with interquartile ranges. Tabular results of differences in the time course of Tfh cells between patient and controls are shown in supplementary table S3. ICOS, inducible T-cell costimulator; PD-1, Programmed cell death protein 1.

## Figure S6


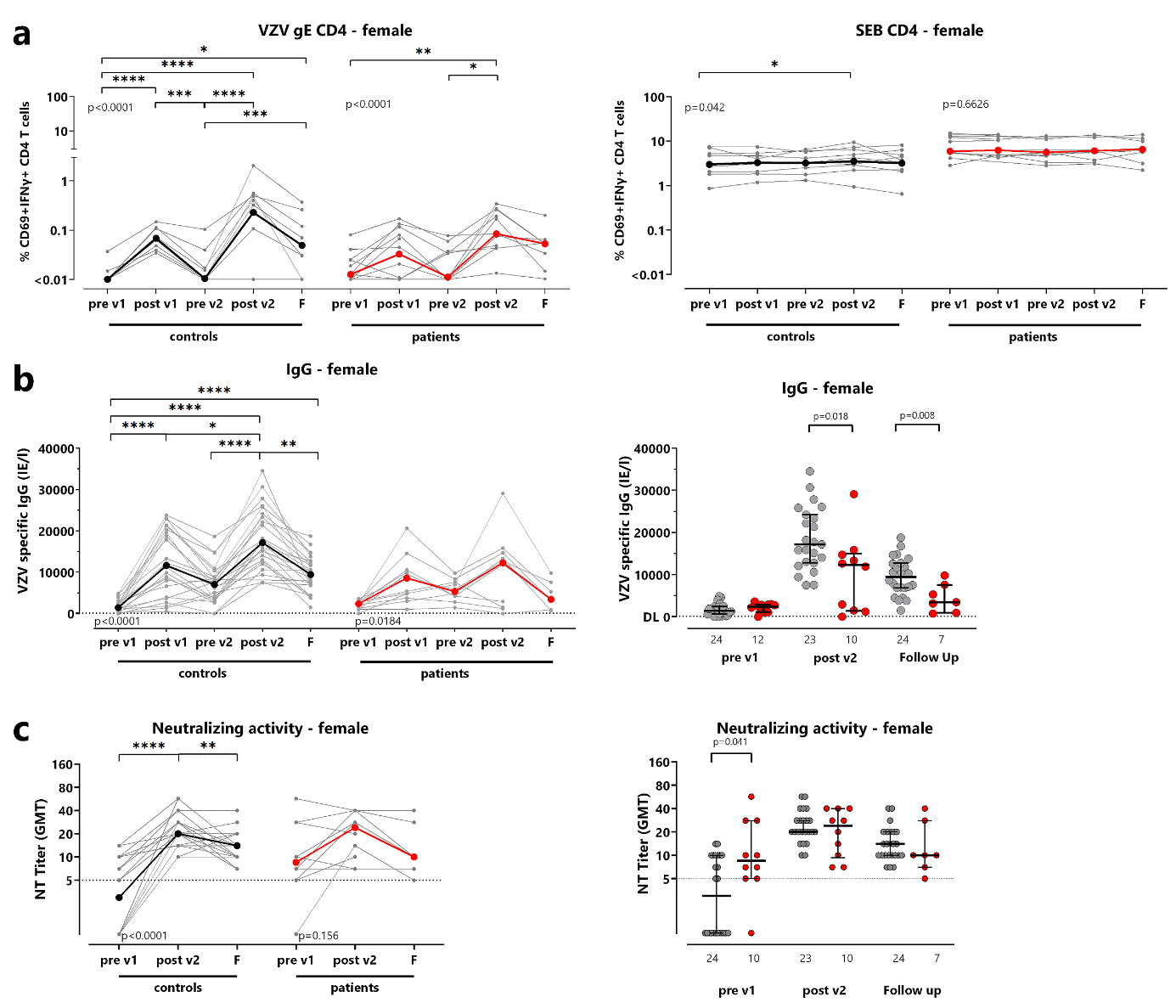


**Figure S6: Immunogenicity in female study participants.** **(a)** Percentages of VZV-specific (left) and SEB-reactive CD4 T cells (right) after subtraction of the corresponding negative control from female controls (black) and patients (red) over time. Bold lines represent median values. Friedman test with Dunn´s post test was performed for statistical analysis. (**b)** VZV-specific IgG levels (in IE/l) from female controls (black) and patients (red) over time (left). Bold lines represent median values. Friedman test with Dunn´s post test was performed for statistical analysis. Comparison of VZV-specific IgGs between controls and patients before first vaccination (pre v1), after second vaccination (post v2) and at follow-up (right). Bars represent median values with interquartile ranges. Statistical analysis was performed using Mann-Whitney test. **(c)** VZV-specific neutralization in female controls (black) and patients (red) over time (left). Bold lines represent median values. Friedman test with Dunn´s post test was performed for statistical analysis. Comparison of neutralizing activity between controls and patients before first vaccination (pre v1), after second vaccination (post v2) and at follow-up (right). Bars represent median values with interquartile ranges. Statistical analysis was performed using Mann-Whitney test.

## Figure S7

**
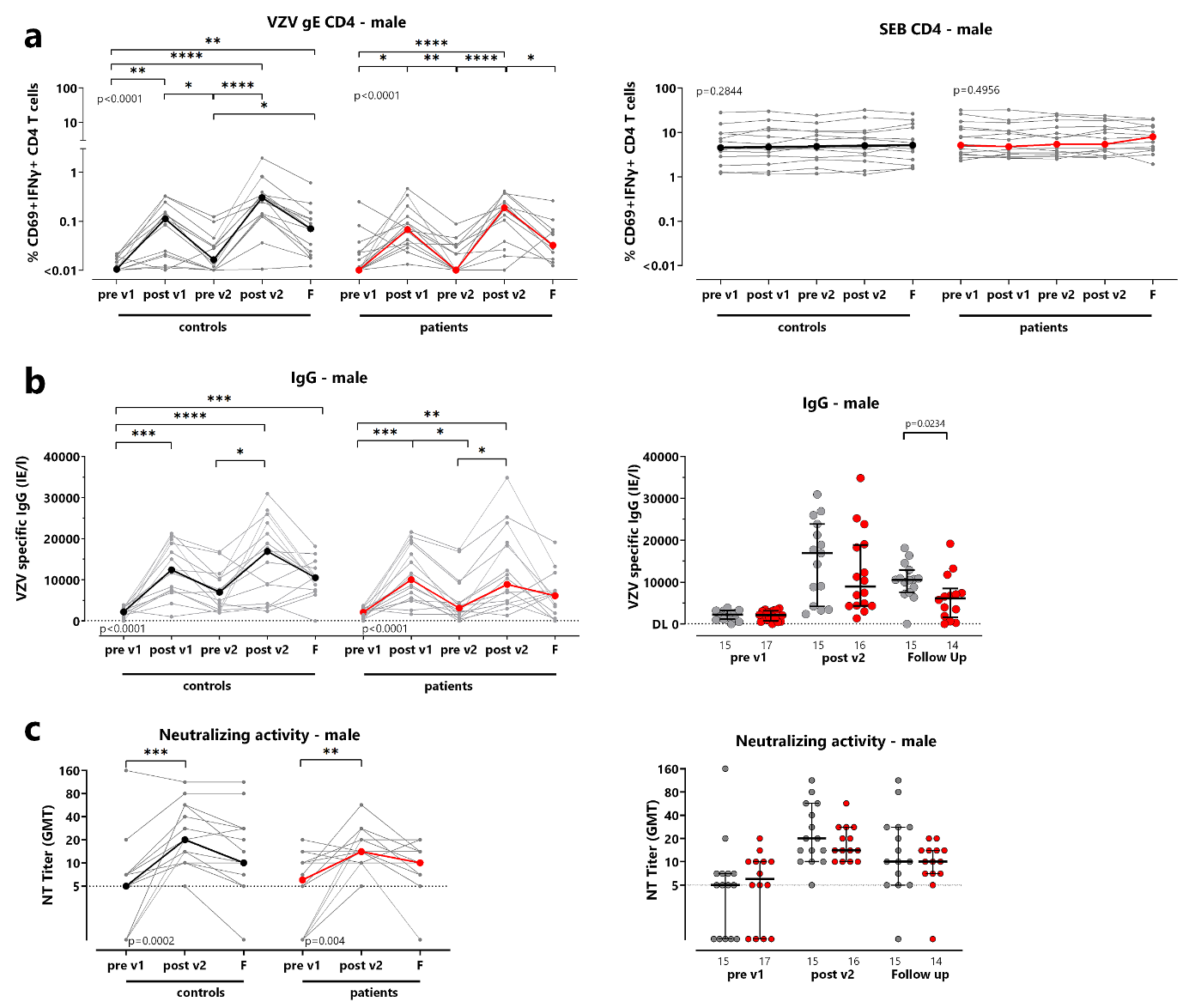
Figure S7: Immunogenicity in male study participants. (a)** Percentages of VZV-specific (left) and SEB-reactive CD4 T cells (right) after subtraction of the corresponding negative control from male controls (black) and patients (red) over time. Bold lines represent median values. Friedman test with Dunn´s post test was performed for statistical analysis. **(b)** VZV-specific IgG levels (in IE/l) from male controls (black) and patients (red) over time (left). Bold lines represent median values. Friedman test with Dunn´s post test was performed for statistical analysis. Comparison of VZV-specific IgGs between controls and patients before first vaccination (pre v1), after second vaccination (post v2) and at follow-up (right). Bars represent median values with interquartile ranges. Statistical analysis was performed using Mann-Whitney test. **(c)** VZV-specific neutralization in male controls (black) and patients (red) over time (left). Bold lines represent median values. Friedman test with Dunn´s post test was performed for statistical analysis. Comparison of neutralizing activity between controls and patients before first vaccination (pre v1), after second vaccination (post v2) and at follow-up (right). Bars represent median values with interquartile ranges. Statistical analysis was performed using Mann-Whitney test.
